# Abstracts from the 3rd Conference on Aneuploidy and Cancer: Clinical and Experimental Aspects: Berkeley, CA, USA. 26 - 29 January 2017

**DOI:** 10.1186/s13039-017-0320-x

**Published:** 2017-06-22

**Authors:** Athel Cornish-Bowden, Athel Cornish-Bowden, David Rasnick, Henry H. Heng, Steven Horne, Batoul Abdallah, Guo Liu, Christine J. Ye, Mathew Bloomfield, Mark D. Vincent, C. Marcelo Aldaz, Jenny Karlsson, Anders Valind, Caroline Jansson, David Gisselsson, Jennifer A. Marshall Graves, Aleksei A. Stepanenko, Svitlana V. Andreieva, Kateryna V. Korets, Dmytro O. Mykytenko, Nataliya L. Huleyuk, Vladimir P. Baklaushev, Oksana A. Kovaleva, Vladimir P. Chekhonin, Yegor S. Vassetzky, Stanislav S. Avdieiev, Bjorn Bakker, Aaron S. Taudt, Mirjam E. Belderbos, David Porubsky, Diana C. J. Spierings, Tristan V. de Jong, Nancy Halsema, Hinke G. Kazemier, Karina Hoekstra-Wakker, Allan Bradley, Eveline S. J. M. de Bont, Anke van den Berg, Victor Guryev, Peter M. Lansdorp, Maria Colomé Tatché, Floris Foijer, Thomas Liehr, Nicolaas C. Baudoin, Joshua M. Nicholson, Kimberly Soto, Isabel Quintanilla, Jordi Camps, Daniela Cimini, M. Dürrbaum, N. Donnelly, V. Passerini, C. Kruse, B. Habermann, Z. Storchová, Daniele Mandrioli, Fiorella Belpoggi, Ellen K Silbergeld, Melissa J Perry, Rolf I. Skotheim, Marthe Løvf, Bjarne Johannessen, Andreas M. Hoff, Sen Zhao, Jonas M. SveeStrømme, Anita Sveen, Ragnhild A. Lothe, R. Hehlmann, A. Voskanyan, A. Fabarius, Alfred Böcking, Stefan Biesterfeld, Leonid Berynskyy, Christof Börgermann, Rainer Engers, Josef Dietz, A. Fritz, N. Sehgal, J. Vecerova, B. Stojkovicz, H. Ding, N. Page, C. Tye, S. Bhattacharya, J. Xu, G. Stein, J. Stein, R. Berezney, Xue Gong, Sarah Grasedieck, Julian Swoboda, Frank G. Rücker, Lars Bullinger, Jonathan R. Pollack, Fani-Marlen Roumelioti, Maria Chiourea, Christina Raftopoulou, Sarantis Gagos, Peter Duesberg, Mat Bloomfield, Sunyoung Hwang, Hans Tobias Gustafsson, Ciara O’Sullivan, Aracelli Acevedo-Colina, Xinhe Huang, Christian Klose, Andrej Schevchenko, Robert C. Dickson, Paola Cavaliere, Noah Dephoure, Eduardo M. Torres, Martha R. Stampfer, Lukas Vrba, Mark A. LaBarge, Bernard Futscher, James C. Garbe, Yi-Hong Zhou, Andrew L. Trinh, Yi-Hong Zhou, Michelle Digman

**Affiliations:** 1 Directeur de Recherche (Émérite), Centre National de la Recherche Scientifique (CNRS), Marseilles, France; 2 Directeur de Recherche (Émérite), Centre National de la Recherche Scientifique (CNRS), Marseilles, France; 3 549 Fairbanks Ave., Oakland, California 94610 USA; 4 Wayne State University School of Medicine, Detroit, MI USA; 5 University of Michigan School of Medicine, Ann Arbor, MI USA; 6 Department of Molecular and Cell Biology, UC Berkeley, Donner Lab, Berkeley, CA USA; 7 Department of Natural Sciences and Mathematics, Dominican University of California, San Rafael, CA USA; 8 Department of Medical Oncology, London Regional Cancer Program, London, Ontario N6A 4L6 Canada; 9 Department of Epigenetics and Molecular Carcinogenesis, University of Texas M.D. Anderson Cancer Center, Science Park, Smithville, TX 78957 USA; 10 Department of Laboratory Medicine, Division of Clinical Genetics, Lund University, Lund, SE221 84 Sweden; 11 Department of Clinical Sciences, Division of Oncology and Pathology, Lund University, Lund, SE221 85 Sweden; 12 Skåne Regional and Univeristy Laboratories, Department of Pathology, Lund, SE221 85 Sweden; 13 School of Life Sciences, La Trobe University, Melbourne, Vic 3168 Australia; 14 Department of Functional Genomics, Institute of Molecular Biology and Genetics, Kyiv, 03680 Ukraine; 15 Institute of Hereditary Pathology, National Academy of Medical Sciences of Ukraine, Lviv, 79008 Ukraine; 16 Federal Research and Clinical Center, FMBA of Russia, Moscow, 115682 Russia; 17 R.E. Kavetsky Institute of Experimental Pathology, Oncology and Radiobiology, Kyiv, 03022 Ukraine; 18 V. P. Serbsky Federal Medical Research Centre of Psychiatry and Narcology, Ministry of Health of the Russian Federation, Moscow, 119034 Russia; 19 Department of Medicinal Nanobiotechnology, Pirogov Russian State Medical University, Moscow, 117997 Russia; 20 CNRS UMR8126, Université Paris-Sud 11, Institut de Cancérologie Gustave Roussy, Villejuif, 94805 France; 21 European Research Institute for the Biology of Ageing, University of Groningen, University Medical Center Groningen, A. Deusinglaan 1, NL-9713 AV Groningen, The Netherlands; 22 Wellcome Trust Sanger Institute, Wellcome Trust Genome Campus, Hinxton, CB10 1SA UK; 23 Department of Paediatrics, University of Groningen, University Medical Center Groningen, A. Deusinglaan 1, NL-9713 AV Groningen, The Netherlands; 24 Department of Pathology & Medical Biology, University of Groningen, University Medical Center Groningen, A. Deusinglaan 1, NL-9713 AV Groningen, The Netherlands; 25 Terry Fox Laboratory, BC Cancer Agency, Vancouver, BC V5Z 1L3 Canada; 26 Department of Medical Genetics, University of British Columbia, Vancouver, BC V6T 1Z4 Canada; 27 Institute for Computational Biology, Helmholtz Zentrum München, Ingolstädter Landstr. 1, 85764 Neuherberg, Germany; 28 Jena University Hospital, Friedrich Schiller University, Institute of Human Genetics, Jena, Germany; 29 Department of Biological Sciences and Biocomplexity Institute, Virginia Tech, Blacksburg, VA 24061 USA; 30 Gastrointestinal and Pancreatic Oncology Group, Institut d’Investigacions Biomèdiques August Pi i Sunyer (IDIBAPS), Hospital Clínic de Barcelona, 08036 Barcelona, Spain; 31 Group Maintenance of Genome Stability, Max Planck Institute of Biochemistry, Martinsried, Germany; 32 Group Computational Biology, Max Planck Institute of Biochemistry, Martinsried, Germany; 33 Department of Molecular Genetics, Technical University Kaiserslautern, Kaiserslautern, Germany; 34 Cesare Maltoni Cancer Research Center, Ramazzini Institute, 40010 Bentivoglio, Bologna, Italy; 35 Department of Environmental Health Sciences, Johns Hopkins Bloomberg School of Public Health, 21205 Baltimore, MD USA; 36 Department of Environmental and Occupational Health, Milken Institute School of Public Health, The George Washington University, 20052 Washington, DC USA; 37 Department of Molecular Oncology, Institute for Cancer Research, Oslo University Hospital, Oslo, Norway; 38 Centre of Cancer Biomedicine, Medical Faculty, University of Oslo, Oslo, Norway; 39 Medizinische Fakultät Mannheim, Universität Heidelberg, Heidelberg, Germany; 40 Institute of Pathology City Hopsital of Düren, Düren, Germany; 41 Institute of Pathology, Koblenz, Germany; 42 Institute of Pathology, Department of Cytopathology, University of Düsseldorf, Düsseldorf, Germany; 43 City Hospital Düren, Department of Urology, Düren, Germany; 44 Institute of Pathology, Lukas-Hospital, Neuss, Germany; 45 Bundesverband Prostatakrebs Selbsthilfe, BPS, Bretten, Germany; 46 College of Medicine, Biochemistry, University of Vermont, Burlington, VT USA; 47 School of Medicine and Biomedical Services, Dermatology, SUNY, Buffalo, NY USA; 48 Department of Biological Sciences, SUNY, Buffalo, NY USA; 49 University at Buffalo, Department of Computer Science and Engineering, SUNY, Buffalo, NY USA; 50 Department of Mathematics and Computer Science, Fayetteville State University, Fayetteville, NC USA; 51 Department of Pathology, Stanford University, Stanford, California 94010 USA; 52 Department of Internal Medicine III, University Hospital of Ulm, Ulm, 89081 Germany; 53 Center for Experimental Medicine and Translational Research, Biomedical Research Foundation of the Academy of Athens Greece (BRFAA), Soranou Efesiou 4, Athens, 115 27 Greece; 54 Department Molecular and Cell Biology UC Berkeley, Donner Lab, Berkeley, CA USA; 55 Department of Natural Sciences and Mathematics, Dominican University, San Rafael, CA USA; 56 Department of Molecular, Cell and Cancer Biology, University of Massachusetts Medical School, Worcester, MA 01605 USA; 57 Department of Molecular and Cellular Biochemistry and the Lucille Markey Cancer Center, University of Kentucky College of Medicine, Lexington, KY 40506 USA; 58 Max Planck Institute of Molecular Cell Biology and Genetics, Dresden, 01307 Germany; 59 Department of Biochemistry, Weill Cornell Medical College, New York, NY 10021 USA; 60 Biological Sciences and Engineering Division, Lawrence Berkeley National Lab, Berkeley, CA 94720 USA; 61 The University of Arizona Cancer Center, Tucson, AZ 85724 USA; 62 Department of Population Sciences, City of Hope, Duarte, CA 91010 USA; 63 Department of Surgery, University of California, Irvine, CA USA; 64 Department of Biomedical Engineering, University of California, Irvine, CA USA; 65 Department of Surgery, University of California, Irvine, CA USA

## I1 Overview of the 3^rd^ Conference on Aneuploidy and Cancer in Berkeley, CA USA in 2017

### (Organized by Peter Duesberg, Department of Molecular and Cell Biology, University of California at Berkeley and David Rasnick, Oakland CA; sponsored by philanthropist Robert Leppo, San Francisco CA USA)

#### Athel Cornish-Bowden (acornish@imm.cnrs.fr)

##### Directeur de Recherche (Émérite), Centre National de la Recherche Scientifique (CNRS), Marseilles, France

David von Hansemann noticed in 1890 that all of the cancer cells that he examined were aneuploid and suggested that the aneuploidy might be the cause of the cancer [1]. Based on Hansemann’s observations and his fundamental discovery that individual chromosomes have different genetic functions, Theodor Boveri advanced the first genetic cancer theory in 1914: cancer is caused by the loss of specific growth inhibitory or the gain of specific growth stimulatory chromosomes [2]. For half a century this remained the prevailing genetic theory of cancer, despite strong opposition from Thomas Morgan, who considered that no instances of chromosomal faults were known to give rise to uncontrolled growth of cells [3]. After the causative agent of Peyton Rous’s chicken sarcoma was discovered to be a retrovirus that inserted an oncogene into the host genome [4], many cancer researchers discarded the aneuploidy theory, considering the dispute to be resolved in favor of oncogenes and viruses. Since then the field has been dominated by the view that cancer is caused by virus-related or virus-unrelated cellular oncogenes.

The difficulty that many researchers had with the chromosomal or aneuploidy theory was that no consistent stimulatory or inhibitory chromosomes could be found in cancers. As a result Boveri’s theory seemed to be stranded on the same reef that prevented the theory of continental drift from being accepted by geophysicists, even though the close fit between Africa and South America had been obvious to anyone who looked at a world map since sufficiently accurate maps became available. In the absence of a credible mechanism, the hypothesis was rejected by most geophysicists, and the fact that Alfred Wegener had no recognized expertise in geophysics doubtless contributed to the skepticism. (Actually, Benjamin Franklin suggested a plausible mechanism similar to the modern theory of plate tectonics as long ago as 1782 [5]).

Returning to cancer, there are also serious difficulties with the oncogene theory. The number of cells in the human body is so large, and the frequency of random mutations in somatic cells is so high, that it is certain that any conceivable oncogene, in its supposedly oncogenic state, is already present in every person, but does not lead to cancer. Such difficulties tend to be brushed aside, just as Wegener’s evidence for continental drift was brushed aside in favor of supposed land bridges that had disappeared. The second problem with oncogenes is that overexpression of single genes almost never has any metabolic effects, and under-expression usually has only minor effects. That is why about 85% of mutations in, say, yeast, are “silent” [6, 7]: eliminating them from the genome usually produces no change in phenotype. When “knock-out mice” first became available [8] it was expected that the function of any gene could be revealed by observing the effect of eliminating it, but most such experiments led to disappointment. When a mouse completely lacks a protein such as myoglobin, which has a well-understood function in muscles, it can remain healthy, even when exercised [9].

The almost negligible effects of altering the activities of single genes can be easily understood in terms of metabolic control analysis, an approach to metabolic regulation introduced by Henrik Kacser and Jim Burns in 1973 [10]. Before then, and for considerable time afterwards, because the new ideas did not become immediately accepted, it was assumed that each metabolic pathway had a “key enzyme” or “rate-limiting enzyme”, and that altering the activity of this enzyme would alter the flux through the pathway in proportion. Kacser and Burns realized, even before many experimental tests were available, that that could not be correct: flux control is shared between all of the enzymes in the system, and if the system is taken to be a whole cell or a whole organism, this means that most shares must be very small, so that altering a typical enzyme activity should have little or no effect. When techniques for genetic manipulation became available, Jürgen Heinisch and colleagues overexpressed phosphofructokinase (widely regarded as the key enzyme in fermentation) in yeast by a factor of 3.5, and observed no effect on the flux to ethanol [11]. This contradicted what was widely expected, but it was not a surprise for people who had understood the principles of metabolic control analysis.

The expectation of no effect, however, is the expectation of what will happen when the activity of a single enzyme is altered. But aneuploidy alters the activities of many enzymes at a time, and the analysis is not so simple. Even in Down syndrome, with fewer than 2% of genes affected by trisomy of the smallest chromosome, the effects are not negligible. Cancer cells are far more aneuploid than those of Down syndrome patients: for example, colon cancer produces cells in which many chromosomes are triploid, some are tetraploid, and some are damaged. This is a vastly larger perturbation than trisomy of one small chromosome, and altering the activities of a vast number of enzymes must inevitably create large metabolic disturbance. Down syndrome illustrates the severe effects that even a minimal degree of aneuploidy can produce, but people who believe that changing a single enzyme activity, or a small number of activities, can lead to cancer need to explain why Down syndrome patients are as normal as they are when they have hundreds of genetic alterations.

I first became conscious of the importance of aneuploidy for cancer when David Rasnick and Peter Duesberg, as well as Harvey Bialy, participated in a meeting that I organized in 1999 [12]. Since then they have used modern concepts of metabolic control as the basis for a quantitative analysis of metabolism in cancer tissue. Their theory goes a long way towards explaining how aneuploidy can be a cause rather than a consequence of cancer. However, there is no longer a reason to doubt that an error in mitosis is the primary event that produces aneuploidy, which eventually leads to cancer. In the first session of the 3rd Conference on Aneuploidy and Cancer, held in Berkeley, California, in January 2017, David Rasnick presented the current state of the theory and compared it with gene-based theories of cancer [13].

This was followed by a lecture in which Henry Heng argued that the chaotic reorganization of the genome that accompanies aneuploidy explains the development of cancer better than a gene- initiated process, and concluded that the survival and adaptive landscapes are different in cancer [14]. Mathew Bloomfield examined the relationships between aneuploidy, karyotypic variability and metastasis, and argued that the appearance of metastases should be regarded as a form of speciation [15]. Mark Vincent continued in this spirit, and discussed why targeted drugs for cancer, with the exception of chronic myeloid leukemia, which he regarded as atypical, have not resulted in the expected benefits. For him carcinogenesis is a form of “de-speciation” rather than speciation [16], but that can be regarded as a matter of definition rather than anything more fundamental. Much later in the conference Peter Duesberg described many problems with gene-based theories, noting, for example, that in contrast to conventional Mendelian genetics no common cancer-specific karyotypes are known; instead, all cancers have individual karyotypes. He argued that carcinogenesis is a type of speciation [17].

Without rejecting the role of aneuploidy, Marcelo Aldaz argued against a Manichean approach to cancer, retaining a role for oncogenes [18]. David Gisselsson described how high-risk cancer is genetically dynamic, both in space and in time [19]. Likewise, Floris Foijer considered that aneuploidy is characteristic of only two out of three tumors (much less than the 100% that David Rasnick reported), and emphasized that p53-deficient knockout mice displayed highly reproducible aneuploidy induction [20]. In a later presentation, Rüdiger Hehlmann discussed the so-called Philadelphia chromosome, which is regularly associated with a definite form of human leukemia. The oncogene ABL on chromosome 9 appears to act in a cooperative process with aneuploidy and development of leukemia. This sort of observation can help to provide new recommendations for the management of acute myeloid leukemia [21]. Jonathan Pollack continued the discussion of this cancer by examining the cryptic genes harbored by complex karyotypes, and described a new computer-based analytical tool for integrating data for copy numbers and gene expression [22].

Thomas Liehr focused on the copy-number variations of chromosomes with no obvious clinical effects, which have been known for decades but have assumed greater visibility with the sequencing of the human genome. He considered that these need more attention in cancer research [23].

The facial cancer that is devastating the population of Tasmanian devils, the largest carnivorous marsupial, is an interesting example in which an oncogene must be completely excluded as a possibility, because it is contagious, as Jennifer Marshall Graves described, and spreads by biting of an affected animal by a healthy one: the cancer itself is transferred. Both the host and the cancer karyotypes are highly conserved; indeed the cancer karyotypes are all the same. She went on to describe the pathogenesis and molecular biology of the tumor [24].

Aleksei Stepanenko returned to the theme of genomic instability as the driving force of cancer, and specifically the adaptation of cancer cells to drugs and transgenic manipulations, arguing that increased resistance to drug re-challenge was the only predictable phenotypic trait observed in all long-term drug-treated tumor cells [25].

Daniela Cimini discussed the view that tetraploidy of the whole genome of a cell can be the precursor of chromosomal instability in cancer, though tetraploidal cell clones with normal centrosome numbers can also occur. She examined the link between aneuploidy and chromosome instability, focusing especially on breast cancer, in which the spontaneous return to an unbalanced diploid cell is accompanied by errors, with monosomy of some chromosomes and damaged fragments of others [26].

Milena Dürrbaum examined the molecular mechanisms underlying aneuploidy in human cells from the point of view of systems biology. She and her colleagues found that gene expression affected common cellular pathways independently of the cell line, type of aneuploidy, and its origin [27].

Daniele Mandrioli described the work of the Ramazzini Institute in Bologna in showing that aneuploidy offered an evidence-based marker for environmental health [28]. (He also pointed out that Theodor Boveri’s theory of cancer [2] was in reality a joint project carried out with his wife, Marcella O’Grady Boveri.)

Rolf Skotheim used computer analysis to study the question of instability of the transcriptome in cancer, with aberrant processing of RNA, specifically in the context of samples of colorectal cancer from 555 patients. They found enormous variation between samples [29].

Alfred Böcking addressed the question of “active surveillance” of localized prostate cancers with a view to avoiding the undesirable side effects that accompany commonly used aggressive therapies, which may well be unnecessary for a substantial proportion of patients, especially in the short term [30].

Andrew Fritz described studies of the spatial arrangement of chromosomes in breast cancer samples, which showed that these arrangements are not random, so that particular “chromosome territories” tend to be associated with particular other ones [31].

Sarantis Gagos discussed the effect of induced replication stress and extreme telomere dysfunction on chromosomal instability and cancer cell stemness. The results from human cell lines with alternative lengthening of telomeres suggested a trend that preserves monoclonality [32].

Eduardo Torres discussed the role of sphingolipids in modulating the fitness of aneuploid cells, because in his view studying the cellular processes affected by aneuploidy can improve our understanding of its role in tumor biology [33]. He concluded that these lipids have important roles in the physiological responses to aneuploidy.

As will be evident from the introductory paragraphs in this report, I am not a great believer in rate-limiting steps in metabolism, and I am not convinced that progression of cancer is exceptional. However, Martha Stampfer took a different view, and argued that immortalization is the rate-limiting step in human carcinogenesis, observing that efficient transformation of normal human mammary epithelial cells does not require gross genomic alterations. She noted that normal cells from small short-lived mammals like mice do not stringently repress telomerase and lack a significant replicative senescence barrier. In consequence, mouse cells are not an adequate model for immortalization in human carcinogenesis [34].

Yi-Hong Zhou studied tumor recurrence after therapy, concluding that tumor heterogeneity could be maintained by missegregation of tumor-specific chromosomes in response to extracellular environmental cues [35]. This interpretation is not necessarily incompatible with Rasnick’s view that recurrence is inevitable after any therapy that destroys only 99.9% of cancer cells, leaving millions (0.1%) to restore a tumor [13], but in any case it is important to understand how tumor heterogeneity is established and maintained. The meeting ended with a presentation by Yi-Hong Zhou’s colleague Michelle Digman, who discussed how fluorescence lifetime imaging microscopy could be used to identify glioblastoma subpopulations [36], which should allow understanding of the role of tumor heterogeneity in drug resistance.

In summary, the 3rd Conference on Aneuploidy and Cancer offered a unique opportunity to discuss many aspects of the origin of aneuploidy and its role in producing cancer—mainly human, but also other organisms such as Tasmanian devils.

**Competing interests**

The author declares no competing interests

**Funding**

The conference and publication were sponsored by philanthropist Robert Leppo, San Francisco CA USA.

**Authors’ contributions**

The author wrote, read and approved the final version of the introduction.

**References**

[1] von Hansemann D. Über asymmetrische Zelltheilung in Epithelkrebsen und deren biologische Bedeutung. *Virchows Arch Pathol Anat*. 1890; 119:299–326.

[2] Boveri T. *Zur Frage der Entstehung maligner Tumoren*. Fisher, Jena 1914.

[3] Morgan TH, Bridges CB. The origin of gynandromorphs. In (ed.) *Contributions to the Genetics of Drosophila melanogaster*. Carnegie Institution, Washington, 1919. pp 108–109.

[4] Duesberg P, Vogt PK. Differences between the ribonucleic acids of transforming and non- transforming avian tumor viruses. *Proc Natl Acad Sci USA*. 1970; 67:1673–1680.

[5] Franklin B. Letter to Abbé Jean-Louis Giraud Soulavie 1782.

[6] Raamsdonk LM, Teusink B, Broadhurst D, Zhang N, Hayes A, Walsh MC, Berden JA, Brindle KM, Kell DB, Rowland JJ, Westerhoff HV, van Dam K, Oliver SG. A functional genomics strategy that uses metabolome data to reveal the phenotype of silent mutations. *Nat Biotechnol*. 2001;19:45–50.

[7] Cornish-Bowden A, Cárdenas ML. Silent genes given voice. *Nature*. 2001;409:571–572.

[8] Capecchi MR. Altering the genome by homologous recombination. *Science*. 1989;244:1288–1292.

[9] Molojavyi A, Lindecke A, Raupach A, Moellendorf S, Köhrer K, Godecke A. Myoglobin-deficient mice activate a distinct cardiac gene expression program in response to isoproterenol-induced hypertrophy. *Physiol Genomics*. 2010;4:137–145.

[10] Kacser H, Burns JA, Fell DA. The control of flux. *Biochem Soc Trans*. 1995;23:341–246. (revision of the paper of 1973).

[11] Schaaff I, Heinisch J, Zimmermann FK. Overproduction of glycolytic enzymes in yeast. *Yeast*. 1989;5:285–290.

[12] Cornish-Bowden A. Metabolic control analysis in biotechnology and medicine. *Nature Biotechnology*. 1999;17:641–643.

[13] Rasnick D. *The chromosomal imbalance theory of cancer: the autocatalyzed progression of aneuploidy is carcinogenesis*: CRC Press, St. Helier 2012. 330p.

[14] Heng H. Debating Cancer: the Paradox in Cancer Research. *World Scientific*. (2015).

[15] Bloomfield, Mathew, and Peter Duesberg. "Inherent variability of cancer-specific aneuploidy generates metastases." *Molecular Cytogenetics* 9.1 (2016): 90.

[16] Vincent MD. Cancer: beyond speciation. *Adv Cancer Res*. 2011;112:283–350.

[17] Duesberg P, Mandrioli D, McCormack A, Nicholson JM. Is carcinogenesis a form of speciation? *Cell Cycle*. 2011;10:2100–2114.

[18] Abba MC, Zhong Y, Lee J, Kil H, Lu Y, Takata Y, Simper MS, Gaddis S, Shen J, Aldaz CM. DMBA induced mouse mammary tumors display high incidence of activating Pik3caH1047 and loss of function Pten mutations. *Oncotarget*. 2016;7:64289–64299.

[19] Mengelbier LH, Karlsson J, Lindgren D, Valind A, Lilljebjorn H, Jansson C, Bexell D, Braekeveldt N, Ameur A, Jonson T, Kultima HG, Isaksson A, Asmundsson J, Versteeg R, Rissler M, Fioretos T, Sandstedt B, Borjesson A, Backman T, Pal N, Ora I, Mayrhofer M, Gisselsson D. Intratumoral genome diversity parallels progression and predicts outcome in pediatric cancer. *Nature Communications*. 2015;6:6125.

[20] Foijer F, Xie SZ, Simon JE, Bakker PL, Conte N, H. Davis SH, Kregel E, Jonkers J, Bradley A, Sorger PK. Chromosome instability induced by Mps1 and p53 mutation generates aggressive lymphomas exhibiting aneuploidy-induced stress. *Proc Natl Acad Sci USA*. 2014;111:13427–13432.

[21] Fabarius A, Leitner A, Hochhaus A, Mueller MC, Hanfstein B, Haferlach C, Goehring G, Schlegelberger B, Jotterand M, Reiter A, Jung-Munkwitz S, Proetel U, Schwaab J, Hofmann W-K, Schubert J, Einsele H, Ho AD, Falge C, Kanz L, Neubauer A, Kneba M, Stegelmann F, Pfreundschuh M, Waller CF, Spiekermann K, Baerlocher GM, Lauseker M, Pfirrmann M, Hasford J, Saussele S, Hehlmann R, Arbeitsgemeinschaft S. German CML Study Group. Impact of additional cytogenetic aberrations at diagnosis on prognosis of CML: long-term observation of 1151 patients from the randomized CML Study IV. *Blood*. 2011;118:6760–6768.

[22] Salari K, Tibshirani R, Pollack JR. DR-Integrator: a new analytic tool for integrating DNA copy number and gene expression data. *Bioinformatics*. 2010;26:414–416.

[23] Liehr T. *Benign and Pathological Chromosomal Imbalances. Microscopic and Submicroscopic Copy Number Variations (CNVs) in Genetics and Counseling*. Academic Press, New York; 2014.

[24] Bender HS, Graves JAM, Deakin JE. Pathogenesis and molecular biology of a transmissible tumor in the Tasmanian devil. In Lewin, H A and Roberts, R M (ed.) *Ann Rev Biosci*. 2014;2:165–187.

[25] Stepanenko AA, Andreieva SV, Korets KV, Mykytenko DO, Baklaushev VP, Chekhonin VP, Dmitrenko VV. mTOR inhibitor temsirolimus and MEK1/2 inhibitor U0126 promote chromosomal instability and cell type-dependent phenotype changes of glioblastoma cells. *Gene*. 2016;579:58–68.

[26] Nicholson JM, Cimini D. Link between aneuploidy and chromosome instability. *Internat Rev Cell Mol Biol*. 2015;315:299–317.

[27] Dürrbaum M, Kuznetsova AY, Passerini V, Stingele S, Stoehr G, Storchova Z. Unique features of the transcriptional response to model aneuploidy in human cells. *BMC Genomics*. 2014;15:139.

[28] Mandrioli D, Belpoggi F, Silbergeld EK, Perry MJ. Aneuploidy: a common and early evidence-based biomarker for carcinogens and reproductive toxicants. *Environmental Health*. 2016;15:97.

[29] Sveen A, Johanssen B, Teixeira MR, Lothe RA, Skotheim RI. Transcriptome instability as a molecular pan-cancer characteristic of carcinomas. *BMC Genomics*. 2014;15:672.

[30] Böcking A, Tils M, Dietz J, Biesterfeld S. DNA-cytometric grading of prostate cancer. Sys- tematic review with descriptive data analysis. *Pathol Discov*. 2014;2:1–20.

[31] Sehgal N, Fritz AJ, Vecerova Ding H, Chen Z, Stojkovic B, Bhattacharya S, Xu J, Berezney R. Large-scale probabilistic 3D organization of human chromosome territories. *Human Mol Genet*. 2016;25:419–436.

[32] Sakellariou D, Chiourea M, C. Raftopoulou C, Gagos S. Alternative lengthening of telomeres: recurrent cytogenetic aberrations and chromosome stability under extreme telomere dysfunction. *Neoplasia*. 2013;15:1301–1313.

[33] Dephoure N, Hwang S, O’Sullivan C, Dodgson SE, Gygi SP, Amon A, Torres EM. Quantitative proteomic analysis reveals posttranslational responses to aneuploidy in yeast. *eLife*. 2013;3:e03023.

[34] Lee JK, Garbe J, Vrba L, Miyano M, Futscher BW, Stampfer MR, LaBarge M. Age and the means of bypassing stasis influence the intrinsic subtype of immortalized human mammary epithelial cells. *Frontiers Cell Develop Biol*. 2015;3:13.

[35] Hu Y, Ru R, Xiao H, Chaturbedi A, Hoa NT, Tian XJ, Zhang H, Chao K, Yan F, Nelson J, Li Z, Gramer R, Yu L, Siegel E, X. Zhang X, Z. Jia Z, M. R. Jadus MR, Limoli CL, Linskey ME, J. Xing J, Zhou YH. Tumor-specific chromosome mis-segregation controls cancer plasticity by maintaining tumor heterogeneity. *PLoS One*. 2013;8(11):e80898.

[36] Trinh A, Zhou YH, Digman MA. Identification of glioblastoma subpopulations by FLIM. Biophys J. 2016;110:651(abstract) http://dx.doi.org/10.1016/j.bpj.2015.11.3482.

## A1 Metabolic control and chromosomal abnormalities

### Athel Cornish-Bowden (acornish@imm.cnrs.fr)

#### Directeur de Recherche (Émérite), Centre National de la Recherche Scientifique (CNRS), Marseilles, France

A theory of what goes wrong with metabolism in cancer requires an adequate theory of what goes right in healthy cells. This needs to be based on the theories of metabolic control and metabolic regulation. To a first approximation the rate of an isolated enzyme-catalysed reaction is proportional to the enzyme concentration, but that is not necessarily the case for multi-enzyme systems, in which flux control is shared among all the enzymes, and the concentration of a particular enzyme can vary substantially with negligible effects on the flux. For any one enzyme the degree of flux control is defined by a flux control coefficient, which is normally never unity, and for systems of many enzymes it is typically immeasurably small. However, this is theory: does it work in practice? One indication that it is correct comes from many failures to increase production of economically valuable metabolic products by overexpressing the enzymes believed to be rate-limiting. Another is the observation that in a genetic disease such as phenylketonuria, heterozygotes with half of the normal amount of enzyme have no symptoms and are just as healthy as normal homozygotes. Likewise, inheritance of eye colour in humans is explained by the fact that eyes with only half the activity of the enzymes needed to produce brown eyes are barely different from the eyes of brown-eyed homozygotes. These examples involve very small numbers of genes, but in chromosomal disturbances that affect many genes, such as Down syndrome or more severe cases of aneuploidy, a large number of effects that would be negligible when considered individually, can add up to very large effects.

The theory of metabolic control needs to be supplemented, however, with a theory of metabolic regulation, to explain why unregulated natural systems are very different in behaviour from living systems or systems designed by engineers. A lake will typically have many sources of water, but a maximum of one exit, whereas engineered and living systems allow regulated flow in different directions. A healthy metabolic system has regulated flows at many different points, and allows forward and reverse transformations between, for example, fructose 6-phosphate and fructose 1,6- bisphosphate, to be possible in the same cells with negligible loss of ATP by hydrolysis. In this example regulation is achieved by strong inhibition and activation of phosphofructokinase and fructose bisphosphatase, to ensure that both processes are not simultaneously in operation. The underlying principle of metabolic regulation by feedback effects is that fluxes are determined primarily by the demand for end-products and as little as possible by the supply of starting materials. If the necessary regulatory interactions are impaired, as they may be in cancer and other illnesses, satisfactory metabolism becomes impossible.

**Further reading**

1. Kacser H, Burns JA, Fell DA. The control of flux. *Biochemical Society Transactions* 1995;23, 341-366. (revised version of the 1973 paper)

2. Cornish-Bowden A. *Fundamentals of Enzyme Kinetics* (4th edition): Wiley-VCH, Weinheim; 2012. Chapter 13.

3. Cornish-Bowden A. (2016) *Biochemical Evolution: the Pursuit of Perfection* (2nd edition): Garland Science, New York; 2012. Chapter 8.

## A2 Competing theories of cancer: chromosomal imbalance vs. gene mutation

### David Rasnick (drasnick@me.com)

#### 549 Fairbanks Ave., Oakland, California, 94610, USA

**Gene mutation theory in decline**

In 2002, Robert Weinberg admitted that, “For those who believe in the simplification and rationalization of the cancer process, the actual course of research on the molecular basis of cancer has been largely disappointing. Rather than revealing a small number of genetic and biochemical determinants operating within cancer cells, molecular analyses of human cancers have revealed a bewilderingly complex array of such factors [1].” In 2014, he added, “Moreover, even within a given type of cancer…there were no uniform successions of genetic change. Instead, each tumor seemed to represent a unique experiment of nature, acquiring a unique set of mutant genes and in an unpredictable chronological order.” Weinberg concluded, “The coupling between observational data and biological insight is frayed if not broken [2].” November 2016, Bert Vogelstein dealt the mutation theory another blow when he reported that the search for cancer causing genes is “hindered by the lack of a gold standard, that is, bona fide driver gene mutations [3].” It was actually a double blow since driver genes include oncogenes and tumor suppressor genes.

**Theory of chromosomal imbalance**

In contrast to “bona fide driver gene mutations,” which are exceedingly difficult to find, aneuploidy is abundant in cancer cells [4]. Chromosomal imbalance is orders of magnitude more powerful than gene mutations in producing cancer phenotypes [5]. The phenotypes of cancer cells are determined by the fraction of the genome out of balance relative to the euploid cell [6]. Aneuploid cancer cells have substantially greater amounts of DNA, RNA and protein than normal cells [7]. A 40% increase in cellular protein produces a 32-fold elevation in membrane proteins. A 20% increase in cellular protein causes a 30-fold elevation in secreted proteins [8]. Thus the tumor-associated antigens and the high levels of secreted proteins responsible for invasiveness and loss of contact inhibition are the natural consequence of the excess production of protein in cancer cells. The additional ATP required for the synthesis of the extra protein is produced by the aerobic fermentation of glucose [9-11], the so-called Warburg effect.

Chromosomal imbalance disrupts the mitotic machinery leading to the chaotic separation of chromosomes. “The relationship between aneuploidy and chromosomal instability can be envisioned as a ‘vicious cycle,’ where one potentiates the other [12].” An extra copy of a single chromosome is sufficient to produce chromosomal instability [13]. The greater the imbalance of chromosomes, the greater is the instability [6, 14]. The survival advantage of cells that gained chromosomes, coupled with chromosomal instability, leads to the autocatalyzed progression of aneuploidy during cell division [6, 15, 16].

**Much sought-after mechanism of carcinogenesis**

Carcinogen-initiated chromosomal imbalance, coupled with the autocatalyzed progression of aneuploidy during cell division, is necessary and sufficient to generate cancer on the rare occasions the cells survive—independent of gene mutation.

**Practical utility**

The theory of chromosomal imbalance has a number of practical applications [17]. Monitoring aneuploidy is the most accurate and sensitive way of detecting cancer and following its progression [18]. Reducing exposure to aneuploidogens is the best way to reduce the incidence of cancer. Aneuploidy damages a cell and is the reason primary cancer cells tend to die at high rates *in vivo* and in culture [19, 20]. Indeed, the lability inherent in aneuploid cells is the likely reason for the spontaneous remission of all types of cancer. The theory of chromosomal imbalance predicts that a variety of non-toxic perturbations of the host (such as induced fever) may therapeutically nudge the tumor out of its stable, comfortable environment, increasing the natural spontaneous death rate of the cancer cells [17].

**References**

1. Hahn WC, Weinberg RA. Rules for making human tumor cells. *N Engl J Med*. 2002;347(20):1593-603.

2. Weinberg RA. Coming full circle-from endless complexity to simplicity and back again. *Cell*. 2014;157(1):267-71.

3. Tokheim CJ, Papadopoulos N, Kinzler KW, Vogelstein B, Karchin R. Evaluating the evaluation of cancer driver genes. *Proc Natl Acad Sci U S A*. 2016;113(50):14330-5.

4. Atkin NB, Huang X. Are human cancers ever diploid - or often trisomic?: an update. *Cytogenet Cell Genet*. 2001;92(3-4):345-6.

5. Duesberg P, Li R, Fabarius A, Hehlmann R. Aneuploidy and cancer: from correlation to causation. *Contrib Microbiol*. 2006;13:16-44.

6. Rasnick D, Duesberg PH. How aneuploidy affects metabolic control and causes cancer. *Biochem J*. 1999;340 (Pt 3):621-30.

7. Caspersson TO. Disturbed systems for protein formation in the metazoan cell. Cell growth and cell function: A cytochemical study. New York: W. W. Norton & Company; 1950. p. 141-51.

8. Minton AP. Influence of macromolecular crowding on intracellular association reactions: possible role in volume regulation. In: Strange K, editor. Cellular and Molecular Physiology of Cell Volume Regulation. Ann Arbor: CRC Press; 1994. p. 181-9.

9. Dolfi SC, Chan LL, Qiu J, Tedeschi PM, Bertino JR, Hirshfield KM, et al. The metabolic demands of cancer cells are coupled to their size and protein synthesis rates. *Cancer & metabolism*. 2013;1(1):20.

10. Vazquez A, Oltvai ZN. Molecular crowding defines a common origin for the Warburg effect in proliferating cells and the lactate threshold in muscle physiology. *PLoS ONE*. 2011;6(4):e19538.

11. Vazquez A, Oltvai ZN. Macromolecular crowding explains overflow metabolism in cells. *Scientific reports*. 2016;6:31007.

12. Potapova TA, Zhu J, Li R. Aneuploidy and chromosomal instability: a vicious cycle driving cellular evolution and cancer genome chaos. *Cancer Metastasis Rev*. 2013;32(3-4):377-89.

13. Passerini V, Storchova Z. Too much to handle - how gaining chromosomes destabilizes the genome. *Cell Cycle*. 2016;15(21):2867-74.

14. Duesberg P, Rausch C, Rasnick D, Hehlmann R. Genetic instability of cancer cells is proportional to their degree of aneuploidy. *Proc Natl Acad Sci U S A*. 1998;95(23):13692-7.

15. Duesberg P, Li R. Multistep carcinogenesis: a chain reaction of aneuploidizations. *Cell Cycle*. 2003;2(3):202-10.

16. Fabarius A, Hehlmann R, Duesberg PH. Instability of chromosome structure in cancer cells increases exponentially with degrees of aneuploidy. *Cancer Genet Cytogenet*. 2003;143(1):59-72.

17. Rasnick D. *The chromosomal imbalance theory of cancer: the autocatalyzed progression of aneuploidy is carcinogenesis*: CRC Press; 2012. 330 p.

18. Böcking A. *With Cells Instead of Scalpels*. 1st ed: Noki d.o.o.; 2012. 112 p.

19. Foulds L. The experimental study of tumor progression: a review. *Cancer Res*. 1954;14(5):327-39.

20. Atkin NB, Baker MC. Are human cancers ever diploid--or often trisomic? Conflicting evidence from direct preparations and cultures. *Cytogenet Cell Genet*. 1990;53(1):58-60.

## A3 Genome chaos and macro-cellular evolution: how chromosomal coding and fuzzy inheritance drive cancer evolution

### Henry H. Heng^1^, Steven Horne^1^, Batoul Abdallah^1^, Guo Liu^1^, Christine J. Ye^2^

#### ^1^Wayne State University School of Medicine, Detroit, MI, USA; ^2^University of Michigan School of Medicine, Ann Arbor, MI, USA

##### **Correspondence:** Henry H. Heng (hheng@med.wayne.edu)

**Background**: Current cancer evolutionary research has been framed by the Gene Mutation Theory and by Neo-Darwinia stepwise evolution. Conventional wisdom includes: 1) cancer is a genetic disease caused by common cancer gene mutations; 2) cancer development follows the process of the accumulation of small genetic changes over time, or clonal evolution; and 3) precision genetic profiling is the key for diagnosis/molecular targeting. Those concepts, however, are challenged by sequencing data and the two phases of cancer evolution (punctuated phase where drastic karyotype changes dominate and stepwise phase where gene mutation and epigenetic alteration dominate). Furthermore, the cytogenetic description of genome chaos, the rapid and massive genome re-organization under stress, and its recent confirmation by cancer genome sequencing call for the search for a new genome based framework to illustrate how cellular systems pass genetic information, and to understand why the karyotype, and not the gene, defines genetic blueprint and is responsible for punctuated cancer evolution.

**Methods**: Using a somatic cellular model, we have traced the pattern of karyotype evolution comparing gene and transcriptome profiling. Specifically, key evolutionary transitions (e.g. immortalization, transformation, metastasis, and drug resistance) were monitored by both single cell and population profiles. Further synthesis was performed to search for novel types of inheritance above gene/epigene and to characterize fuzziness for chromosomal or karyotype coding. [1]

**Conclusion**: Data analysis and their synthesis with the genome theory have led to the following conclusions: 1) chromosome or karyotype codes for the address of genes within the nuclei. Such system inheritance differs from gene-coded “parts inheritance”, defining the network structure. Most cancers belong to different genome defined systems; 2) genomic coding is less precise; rather, it is fuzzy. Fuzzy inheritance defines a range of genetic information but not a fixed status, which is the mechanism of inherited heterogeneity. Since the degree of heterogeneity is inherited, a single cell can restore the cell population heterogeneity, and non clonal chromosome aberrations (NCCAs) are common in cancer representing population potential; 3) heterogeneity or “noise” defines cancer, and the outliers are highly significant in cancer evolution. Heterogeneity can also be generated from cellular adaptive processes, and thus represent an unavoidable feature. Genome or karyotype heterogeneity can be rapidly achieved by genome chaos including massive fusion/dividing, and possibly by the requirement of minimal survivable information packages. Furthermore, the transitional population can be achieved by the local cellular or tumor society where individual cells are not sufficient enough to independently survive (but the emergence of mixed cellular populations can); 4) the evolutionary mechanism of cancer unifies the diverse molecular mechanisms. The multiple level landscape model explains the limitations of focusing on cancer pathway studies; and 5) the key cancer transition is mainly achieved by new genome-mediated-macro-cellular emergence, while gene mutations are mainly responsible for system modification. Survival and adaptive landscapes differ in cancer, which suggests the importance of separating macro and micro-cellular evolution [1-7].

**References**

1. Heng HH (2015) Debating Cancer: The Paradox in Cancer Research, World Scientific Publishing, New Jersey.

2. Heng HH, Regan SM, Liu G, Ye CJ. (2016). Why it is crucial to analyze non clonal chromosome aberrations or NCCAs? Mol Cytogenet, 9: 15.

3. Heng HH (2017) Genomic landscape of cancer, in Ecology and Evolution of Cancer, eds, Beata Ujvari B, Roche B, Thomas F, Academic Press, pp 69-86.

4. Horne SD, Chowdhury SK, Heng HH. (2014). Stress, genomic adaptation, and the evolutionary trade-off. Front Genet, 5.92: 1-6.

5. Horne SD, Ye CJ, Heng HH. (2015). Chromosomal instability (CIN) in cancer. eLS, 1-9. doi/10.1002/9780470015902.a0006069.pub2/full

6. Liu G, Stevens JB, Horne SD, Abdallah BY, Ye KJ, Bremer SW, Ye CJ, Chen DJ, Heng HH. (2014). Genome chaos: Survival strategy during crisis. Cell Cycle, 13.4: 528-537.

7. Stevens JB, Liu G, Abdallah BY, Horne SD, Ye KJ, Bremer SW, Ye CJ, Krawetz SA, Heng HH. (2014). Unstable genomes elevate transcriptome dynamics. Int J Cancer, 134.9: 2074-2087.

## A4 Why metastasis risk is proportional to the degree of cancer-specific aneuploidy

### Mathew Bloomfield^1,2^ (mbloomfield@berkeley.edu)

#### ^1^Department of Molecular and Cell Biology, UC Berkeley, Donner Lab, Berkeley, CA, USA; ^2^Department of Natural Sciences and Mathematics, Dominican University of California, San Rafael, CA, USA

**Background**

Metastasis accounts for nearly 90% of cancer-related mortality, but a genetic mechanism remains elusive [1]. Previous research indicates that metastasis risk increases with the DNA content of the primary cancer [2-11]. However, a coherent theory is still needed to explain metastasis [1, 12-13].

**Carcinogenesis as a form of speciation**

We and others have recently developed a theory that considers the karyotypic origin of carcinogenesis, namely the speciation theory. This theory contends that cancer is generated by random karyotypic variations and evolutions of aneuploid cells from which a rare, autonomous cancer cell emerges at aneuploidy-dependent rates [14]. The theory further predicts that cancers progress or evolve by automatic karyotypic variations, because aneuploidy unbalances thousands of genes—the more aneuploid the cancer the more likely it forms a metastatic variant [14, 15].

Here we test the prediction of our theory that cancer-specific aneuploidy catalyzes the karyotypic evolution of metastatic variants from primary cancer.

Specifically, we asked:

1) Do primary cancers and corresponding metastases have related yet individual karyotypes?

2) Does the degree of cancer-specific aneuploidy enhance the range of cancer-specific karyotypic variation and by inference the risk of metastasis?

**Results**

To answer these questions, twenty single metaphases from each primary cancer were compared to determine how cancer-specific aneuploidy affects karyotypic variability. We found that all cancers and corresponding metastases had individual clonal, or quasi-clonal, karyotypes by methods we have described recently [15]. Moreover, all metastases shared clonal aneusomies with parental cancers, but also contained individual clonal aneusomies.

Next, we asked how the degree of aneuploidy of different primary cancers affected the degree of aneuploidy of the corresponding metastases. In these comparative studies, we found that the karyotypic heterogeneity of near-diploid breast cancer HIM-2 was low, averaging only 1.1 non-clonal aneusomies per 20 metaphases. Accordingly, the corresponding metastasis HIM-5 also had a near-diploid karyotype with a similarly low level of karyotypic heterogeneity.

By contrast, the karyotypic heterogeneity of hyper-triploid medulloblastoma M-458, the hyper-triploid liver cancer H2M, and the hypo-triploid pancreatic cancer A13-B was high, averaging 17.6, 11.45, and 10.15 non-clonal aneusomies per 20 metaphases, respectively. Likewise, their metastases also had hypo-triploid and hyper-triploid karyotypes and similarly high levels of karyotypic heterogeneity. These results indicate that cancer-specific karyotypic heterogeneity, and thus inherent variability, is dependent on cancer-specific aneuploidy.

In sum, our results showed that:

1) All metastases tested shared specific clonal aneusomies with the corresponding primary cancers, but differed from parental cancers in individual aneusomies as well. This confirms the prediction of our theory that metastases are sub-clones (or subspecies) from primary cancers.

2) The degree of aneuploidy of primary cancers was inversely proportional to the karyotypic relationship between their metastases. This confirms the prediction of our theory that the degree of aneuploidy determines the degree of clonal heterogeneity, and thus the inherent variability of cancers.

**Conclusions**

We conclude that cancers with a high degree of aneuploidy are highly heterogeneous within cancer-specific margins due to high karyotypic variability. Highly aneuploid cancers are thus at greater risk of metastasizing than cancers with little aneuploidy. Therefore, the degree of cancer-specific aneuploidy and, particularly, karyotypic variability are key markers of metastasis risk.

**Acknowledgements**

I’d like to acknowledge Peter Duesberg (UC Berkeley) for critical review and mentorship during this project and Peter Walian (Lawrence Berkeley Laboratory) for helpful comments and discussion. Bob Leppo (San Francisco) is specifically thanked for his philanthropic support, which made this research and conference possible.

**References**

1. Weinberg RA: The biology of cancer, Second edition. edn. New York, NY; London: Garland Science; 2014.

2. Atkin NB: Modal deoxyribonucleic acid value and survival in carcinoma of the breast. *Br Med J* 1972, 1(5795): 271-272.

3. Frankfurt OS, Chin JL, Englander LS, Greco WR, Pontes JE, Rustum YM. Relationship between DNA ploidy, glandular differentiation, and tumor spread in human prostate cancer. Cancer Res. 1985;45(3):1418–23.

4. Wolman SR. Cytogenetic heterogeneity: its role in tumor evolution. Cancer Genet Cytogenet. 1986;19(1–2):129–40.

5. Ljungberg B, Stenling R, Roos G. Prognostic value of deoxyribonucleic acid content in metastatic renal cell carcinoma. J Urol. 1986;136(4):801–4.

6. Fallenius AG, Franzen SA, Auer GU. Predictive value of nuclear DNA content in breast cancer in relation to clinical and morphologic factors. A retrospective study of 227 consecutive cases. Cancer. 1988;62(3):521–30.

7. Saito T, Sato J, Satoh A, Notani K, Fukuda H, Mizuno S, Shindoh M, Amemiya A. Flow cytometric analysis of nuclear DNA content in tongue squamous cell carcinoma: relation to cervical lymph node metastasis. Int J Oral Maxillofac Surg. 1994;23(1):28–31.

8. Hemmer J, Thein T, Van Heerden WF. The value of DNA flow cytometry in predicting the development of lymph node metastasis and survival in patients with locally recurrent oral squamous cell carcinoma. Cancer. 1997;79(12):2309–13.

9. Torres L, Ribeiro FR, Pandis N, Andersen JA, Heim S, Teixeira MR. Intratumor genomic heterogeneity in breast cancer with clonal divergence between primary carcinomas and lymph node metastases. Breast Cancer Res Treat. 2007;102(2):143–55.

10. Haugvik SP, Gorunova L, Haugom L, Eibak AM, Gladhaug IP, Heim S, Micci F. Loss of 11p11 is a frequent and early event in sporadic nonfunctioning pancreatic neuroendocrine neoplasms. Oncol Rep. 2014;32(3):906–12.

11. Jonkers YM, Claessen SM, Perren A, Schmitt AM, Hofland LJ, de Herder W, de Krijger RR, Verhofstad AA, Hermus AR, Kummer JA, et al. DNA copy number status is a powerful predictor of poor survival in endocrine pancreatic tumor patients. Endocr Relat Cancer. 2007;14(3):769–79.

12. Foulds L: Neoplastic Development, vol. 2. London, New York, San Francisco: Academic Press; 1975.

13. Nowell PC: The clonal evolution of tumor cell populations. *Science* 1976, 194(4260):23-28.

14. Bloomfield M, McCormack A, Mandrioli D, Fiala C, Aldaz CM, Duesberg P: Karyotypic evolutions of cancer species in rats during the long latent periods after injection of nitrosourea. *Mol Cytogenet* 2014, 7(1):71.

15. Bloomfield M, Duesberg P: Inherent variability of cancer-specific aneuploidy generates metastases. *Mol Cytogenet* 2016, 9:90.

## A5 On the nature of cancer, and why it matters

### Mark D. Vincent (Mark.Vincent@lhsc.on.ca)

#### Department of Medical Oncology, London Regional Cancer Program, London, Ontario, N6A 4 L6, Canada

According to conventional wisdom, cancer is a disease process, arising from genetic errors in the command and control apparatus of a precursor cell. Unregulated clonal expansion coupled with additional genetic errors then follows, and, by Darwinian selection, the tumor evolves to a more aggressive (and drug-resistant) phenotype. This is the somatic mutation theory (‘M-Theory’). Accordingly, the solution to the problem of clinical cancer requires the description of the molecular events mediating escape from growth constraints. This paradigm depends on notions of causality and explanation, leading inevitably to the concept of molecularly targeted therapy to achieve both the required efficacy and the necessary selectivity; it also involves the belief that such targets must exist, and are tractable.

Technical progress in genomic sequencing seemed to promise the realization of this dream, but despite the torrent of big data, the clinical utility has disappointed; few cancers possess truly ‘actionable mutations’ and, except for chronic myeloid leukemia (an atypically simplified cancer), the benefits of modern targeted drugs (while undeniable) are transient, usually lasting about one year only. M-Theory has not delivered, and given the inherent and extensive obstacles, may never deliver [1]. Indeed, it now faces a Kuhnian crisis portending ‘model revolution’. Unfortunately for causality-focused theorists, no deeper level of drill-down exists beyond next-generation sequencing, and hence no obvious alternative routes to a better causality-based model.

An alternative model re-focuses on the cancer cell as an organism per se, a different form of life from the host. Malignant transformation is seen as speciation, but a form of speciation that is radically different from the usual allopatric speciation generally accounting for the peripheral arborization of the tree of life. In this alternative view, the cancer cell is not another type of metazoan closely related to our phenotype, but in fact a protozoan, and not just an ordinary protozoan; it is uniquely adapted to the very different geochemistry of the ancient Proterozoic eon (circa 2.5BYA – 0.514MYA) via a stereotypical set of peculiar characteristics common to all cancers.

The very commonality of these characteristics of the malignant phenotype, in the face of so much genomic heterogeneity, is actually a weakness of M-Theory which can only ascribe it to convergent evolution; but this is not parsimonious, given the requirement that every cancer throughout history and in every type of host is then obliged to re-invent the same wheel each time. The more parsimonious explanation is not M-Theory’s convergent evolution, but the unmasking of a common inheritance dating back to the emergence of the eukaryotic cell some 1.6BYA, in the hypoxic, acidotic and sulfurous oceans of the mid-Proterozoic. This is atavism (‘A-Theory’), in which the in-common traits of the cancer cell are either primitive (perhaps including aneuploidy, ie species fluidity) or adaptations to the then contemporaneous environment (e.g. Warburg Effect, proton pump) of the original early eukaryote.

Therapeutic implications here depend not on causality-reversal, but on the principle of recognition, in which cancer treatment results in the release of destructive forces acting selectively on the cancer cell, mediated by signature-attributes (including large-scale genomic alteration). Two illustrative ascendant examples are therapeutic DNA-repair disruption (selectively pushing the cancer cell beyond viability) and treatment with immune checkpoint inhibitors (selectively unleashing the immune system on the neoantigen-painted cancer cell, now seen as a foreign, ultimately because the deranged genome manufactures an altered, hence antigenic proteome).

The concept of the target is thus expanded beyond the causal mediators of the malignant phenotype, to include a variety of signature differences which might have a common explanation in deep time.

**References**

1. Tannock IF and Hickman JA. Limits to precision cancer medicine. *N Engl J Med* 2016; 376: 96-97

## A6 Aneuploidy in premalignant progression: lessons from experimental carcinogenesis models to human Ductal Carcinoma in situ (DCIS)

### C. Marcelo Aldaz (maaldaz@mdanderson.org)

#### Department of Epigenetics and Molecular Carcinogenesis, University of Texas M.D. Anderson Cancer Center, Science Park, Smithville, TX 78957, USA

**Important concepts**

1. *Manichean definition: Manichean comes from the word Mani, which is the name of an apostle who lived in Mesopotamia in the 240’s, who taught a universal religion based on what we now call dualism. If you believe in the Manichean idea of dualism, you tend to look at things as having two sides that are opposed. To Manicheans, life can be divided neatly between good or evil, light or dark, love and hate, right and wrong. So if your thinking is Manichean, you see things in black and white.* 2. *“Nothing is black or white” Nelson Mandela*. 3. *“Don't reinvent the wheel, just realign it” Anthony J. D’Angelo*.

It is worth noting that a simple search in PubMed with the words ‘aneuploidy cancer’ retrieves 17,740 publications and if we limit this search to ‘Reviews’ one retrieves 2,257 publications as of 12/2016. In other words we are not dealing with a new issue or revolutionary concepts, nor we are dealing with a topic that is ‘white or black’ i.e. somatic mutation vs. aneuploidy (or chromosomal instability). Everything that could have been said about the relevance of aneuploidy and somatic mutation in cancer initiation and progression has already been proposed and written…long ago. There is no Holy Grail or ultimate truth. The truth is never black or white is ‘gray’. Beating a death horse on either side will take us nowhere and is truly irrelevant.

Progressive aneuploidy development is important in cancer initiation and progression and so are mutations in cancer driver genes and so is the Darwinian fitness of clones (not a new concept by ‘any’ means). Experimental and observational evidence indicates that there is a clear synergy between these phenomena. Who comes first? The answer (if a single one exists) will have little if ‘any’ public health impact.

It is clear from decades old studies, by us and others, that somatic mutations in cancer driver genes (e.g. Ha-Ras) are real, and are induced by carcinogens (e.g. DMBA) at ‘sub-carcinogenic doses’ in other words at doses in which no tumors develop during the lifespan of the mouse. If the tissues (e.g. skin) bearing mutated clones are exposed to ‘tumor promoter stimuli’ (e.g. TPA) that induces protracted inflammation and proliferation with consequential ‘random’ aneuploidy development. Eventually non-random trisomies (trisomies of chr. 6 and 7) develop in premalignant lesions (papillomas). Importantly, there is a selection for clones bearing trisomized chr. 7 carrying mutated Ha-Ras allele with loss of Ha-Ras wt. alleles. In summary, no promotion = no aneuploidy = no tumors, but also sub-carcinogenic DMBA = no tumors, even in the presence of promotion [1-4].

My laboratory demonstrated very similar results in mammary cancer models in which duplications trisomies and amplification affecting the mutated Ha-Ras locus in rat chromosome 1 are observed upon progression from hormone dependent tumors to hormone independency. In this case the ‘promoter’ are the ovarian produced hormones [5-6]. Other models will be briefly discussed [7-8].

Finally, in recent studies using Next Generation Sequencing approaches we determined that ‘pure’ Ductal Carcinoma in situ lesions (premalignant by definition), already display mutations, transcriptome and epigenetic changes indistinguishable from invasive breast cancer. Importantly however >80% of pure DCIS lesion display evidence of aneuploidy and in some cases with absence of cancer driver mutations, suggesting that aneuploidy may precede mutation in some cases [9].

Humans are constantly exposed to a myriad of environmental ‘mutagens’ and we are also exposed to endogenous and exogenous ‘promotional stimuli’ that induce proliferation and stimulate aneuploidy development e.g. estrogens, progesterone, androgens, hormone replacement therapy, chronic inflammation etc. It is the synergy of these insults, their consequences and how to minimize them what matters, not what happens first.

**References**

1. Aldaz CM, Conti CJ, Klein-Szanto AJ, Slaga TJ. Progressive dysplasia and aneuploidy are hallmarks of mouse skin papillomas: relevance to malignancy. Proc Natl Acad Sci U S A. 1987;84(7):2029-32

2. Aldaz CM, Trono D, Larcher F, Slaga TJ, Conti CJ. Sequential trisomization of chromosomes 6 and 7 in mouse skin premalignant lesions. Mol Carcinog. 1989;2(1):22-6

3. Bianchi AB, Aldaz CM, Conti CJ. Nonrandom duplication of the chromosome bearing a mutated Ha-ras-1 allele in mouse skin tumors. Proc Natl Acad Sci U S A.1990;87(17):6902-6.

4. Bianchi AB, Navone NM, Aldaz CM, Conti CJ. Overlapping loss of heterozygosity by mitotic recombination on mouse chromosome 7 F1-ter in skin carcinogenesis. Proc Natl Acad Sci U S A. 1991;88(17):7590-4.

5. Aldaz CM, Chen A, Gollahon LS, Russo J, Zappler K. Nonrandom abnormalities involving chromosome 1 and Harvey-ras-1 alleles in rat mammary tumor progression. Cancer Res. 1992;52(17):4791-8.

6. Aldaz CM, Gollahon LS, Chen A. Systematic HRAS amplification in ovary-independent mammary tumors: correlation with progressively anaplastic phenotypes. Cancer Res. 1993;53(22):5339-44.

7. Donehower LA, Godley LA, Aldaz CM, Pyle R, Shi YP, Pinkel D, Gray J, Bradley A, Medina D, Varmus HE. Deficiency of p53 accelerates mammary tumorigenesis in Wnt-1 transgenic mice and promotes chromosomal instability. Genes Dev. 1995;9(7):882-95.

8. Abba MC, Zhong Y, Lee J, Kil H, Lu Y, Takata Y, Simper MS, Gaddis S, Shen J, Aldaz CM. DMBA induced mouse mammary tumors display high incidence of activating Pik3caH1047 and loss of function Pten mutations. Oncotarget. 2016 Aug 31. doi:10.18632/oncotarget.11733. [Epub ahead of print]

9. Abba MC, Gong T, Lu Y, Lee J, Zhong Y, Lacunza E, Butti M, Takata Y, Gaddis S,Shen J, Estecio MR, Sahin AA, Aldaz CM. A Molecular Portrait of High-Grade Ductal Carcinoma In Situ. Cancer Res. 2015;75(18):3980-90.

## A7 A geography of death: mapping clonal evolution over anatomic space in children with cancer

### Jenny Karlsson^1^, Anders Valind^1^, Caroline Jansson^1^, David Gisselsson^1,2,3^

#### ^1^Department of Laboratory Medicine, Division of Clinical Genetics, Lund University, Lund, SE221 84, Sweden; ^2^Department of Clinical Sciences, Division of Oncology and Pathology, Lund University, Lund, SE221 85, Sweden; ^3^Skåne Regional and Univeristy Laboratories, Department of Pathology, Lund, SE221 85, Sweden

##### **Correspondence:** David Gisselsson (david.gisselsson_nord@med.lu.se)

**Aims**

To chart genetic intratumour diversity in childhood cancer over multiple anatomic locations and/or time points during treatment in order to (1) delineate common routes of cancer cell evolution, (2) reveal candidate mechanisms behind treatment resistance, and (3) gain information on intrapatient variability of clinical biomarkers.

**Materials and methods**

Patients were included based on the availability of two or more informative samples from the primary tumour, taken with a minimum intersample distance of 10 mm. A total of 50 patients with Wilms tumour (n = 20), neuroblastoma (n = 20), or sarcoma (n = 10) have so far been subjected to multiregional analysis of tumour tissue with high resolution whole genome genotyping arrays (all patients) complemented in selected cases by whole exome sequencing, targeted deep DNA sequencing, and RNA sequencing. Between two and 20 tumour samples were analyzed per patient with a total of 229 informative tumour samples genotyped so far.

**Results**

The majority of cases exhibited intratumour genetic diversity with branching evolution, including variability of several suggested clinical biomarkers. Subclones were the major arena of genome evolution in most primary tumors. There were clear features of convergent evolution, including trisomies and monosomies of whole chromosomes as well as specific somatic gene alterations such as *TP53* mutation in Wilms tumors, *CDKN2A/B* deletion in neuroblastoma, and *CDK4* amplification in rhabdomyosarcoma. Genetic patterns unique to anatomic sub-compartments in individual patients had functional consequences for gene expression at the RNA and protein levels. So far, four general trajectories of tumour evolution have been identified of which at least one strongly correlates to treatment resistance.

**Conclusion**

Even in very young patients, high-risk cancer is a genetically dynamic disease over space and time. Intratumour genetic diversity is common and is a significant source of error in biomarker determination. Certain evolutionary patterns may be useful as future clinical predictors.

## A8 Chromosomes of the transmissible tumour decimating the Tasmanian devil

### Jennifer A. Marshall Graves (J.Graves@latrobe.edu.au)

#### School of Life Sciences, La Trobe University, Melbourne Vic 3168, Australia

The iconic Tasmanian devil is the largest extant marsupial carnivore. It is confined to the Australian island state of Tasmania, and is an important part of the island’s ecology, as well as a significant tourist attraction. Devils with gross facial tumours were first noticed in north east Tasmania in 1995, and this condition spread over the subsequent decade. Devil Facial Tumour Disease (DFTD) affects the mouth, nose and eyes, and transcriptome analysis suggests a Schwann cell origin [1]. DFTD is invariably and rapidly fatal even before significant metastasis [2]; it has now killed 90% of devils and there is concern about extinction in the wild.

Chromosome analysis first showed that this tumour was transmissible. The devil has a very conserved (almost ancestral marsupial) karyotype of 2n = 14 shared by many species in the Family Dasyuridae. Karyotypes of tumour cells were very abnormal – but notably they were all the same, suggesting that a devil tumour cell itself is the pathogen [3], and is transmitted between adult animals by biting during feeding and mating. This clonal theory was supported by the finding that tumour cells from different animals had the same microsatellite profile and MHC type, which was different from those of host animals.

Chromosome painting revealed that two chromosomes of the tumour karyotype were multiply rearranged, suggesting a chromothrypsis event [4]. The X and chromosome 1 (misidentified as 2 in the first and subsequent DFTD papers) were fragmented and bits inserted at several locations. The absence of Y chromosome sequences, and heterozygosity of some X-borne genes implies that the founder animal was a female. The tumour karyotype is remarkably stable. However, over the last few years, several minor structural variants have been identified, as well as a tetraploid strain [5], and there is some suggestion of differences in transmissability. However, no sign of attenuation has been reported.

Remarkably for such a rare condition, an unrelated transmissible facial tumour (DFTD2) has recently been detected in animals in southern Tasmania [5]. This may relate to the paucity of MHC variation in the devil population and ferocious devil social interactions, or could reflect an unusual imprinted telomere regulation in devils and their dasyurid relatives [6]. A difference between long telomeres on the paternal chromosome set and short on the maternal set is maintained throughout life, but is obliterated in the tumour cells.

**References**

1. Murchison EP, Tovar C, Hsu A, Bender HS, Kheradpour P, et al. (2010) The Tasmanian devil transcriptome reveals Schwann cell origins of a clonally transmissible cancer. Science 327: 84–87

2. Bender, H.S., Graves, J.A.M. and Deakin, J.E. 2014. Pathogenesis and molecular biology of a transmissible tumour in the Tasmanian devil. *Ann. Rev. Animal Biosciences* 2: 165-187

3. Pearse A-M, Swift K (2006) Allograft theory: transmission of devil facial-tumour disease. Nature 439: 549

4. Deakin JE, Bender HS, Pearse A-M, Rens W, O’Brien PCM, et al. (2012) Genomic restructuring in the tasmanian devil facial tumour: chromosome painting and gene mapping provide clues to evolution of a transmissible tumour. PLoS Genet 8: e1002483

5. Pearse A-M, Swift K, Hodson P, Hua B, McCallum H, et al. (2012) Evolution in a transmissible cancer: a study of the chromosomal changes in devil facial tumor (DFT) as it spreads through the wild Tasmanian devil population. Cancer Genet 205: 101–112

6. Bender HS, Murchison EP, Pickett HA, Deakin JE, Strong MA, et al. (2012) Extreme telomere length dimorphism in the Tasmanian devil and related marsupials suggests parental control of telomere length. PLoS ONE 7: e46195.

## A9 Genomic instability drives adaptation of tumor cells to drugs and transgenic manipulations

### Aleksei A. Stepanenko^1^, Svitlana V. Andreieva^1^, Kateryna V. Korets^1^, Dmytro O. Mykytenko^1^, Nataliya L. Huleyuk^2^, Vladimir P. Baklaushev^3^, Oksana A. Kovaleva^4^, Vladimir P. Chekhonin^5,6^, Yegor S. Vassetzky^7^, Stanislav S. Avdieiev^1^

#### ^1^Department of Functional Genomics, Institute of Molecular Biology and Genetics, Kyiv 03680, Ukraine; ^2^Institute of Hereditary Pathology, National Academy of Medical Sciences of Ukraine, Lviv 79008, Ukraine; ^3^Federal Research and Clinical Center, FMBA of Russia, Moscow 115682, Russia; ^4^R.E. Kavetsky Institute of Experimental Pathology, Oncology and Radiobiology, Kyiv 03022, Ukraine; ^5^V. P. Serbsky Federal Medical Research Centre of Psychiatry and Narcology, Ministry of Health of the Russian Federation, Moscow 119034, Russia ; ^6^Department of Medicinal Nanobiotechnology, Pirogov Russian State Medical University, Moscow 117997, Russia; ^7^CNRS UMR8126, Université Paris-Sud 11, Institut de Cancérologie Gustave Roussy, Villejuif 94805, France

##### **Correspondence:** Aleksei A. Stepanenko (a.a.stepanenko@gmail.com)

**Background**

Genomic instability produces genetic and phenotypic heterogeneity that allows a cancer cell population to adapt to and survive harsh microenvironments. Cytotoxic stresses including drug treatment-mediated stress and transgenic manipulations may intensify genomic chaos of tumor cells and favor the emergence of new phenotype variants, increasing the evolutionary potential of a tumor [1,2]. To provide evidence of karyotype-phenotype evolution as an adaptive response to stressful conditions, we characterized chromosomal instability (CIN, clonal and non-clonal chromosomal aberrations, CCAs/NCCAs) and phenotypic changes of cancer cell lines after applying the different cytotoxic stresses.

**Materials and methods**

A panel of the long-term temozolomide, temsirolimus, and U0126-treated U251, T98G, and C6 glioblastoma cell lines was established. HEK293_pcDNA3.1, HEK293_*CHI3L1* and HeLa_*CHI3L1* cell lines were derived by stable transfection with pcDNA3.1 empty plasmid or tumor-associated *CHI3L1* cDNA in pcDNA3.1 vector. Genomic and phenotypic changes were analyzed by conventional cytogenetics, array CGH, cell viability assay, trypan blue exclusion assay, soft agar colony formation assay, scratch wound healing assay, transwell invasion assay, quantitative polymerase chain reaction, and Western blotting.

**Results**

A stable transfection of either pcDNA3.1 or pcDNA3.1_*CHI3L1* into HEK293 cells or HeLa cells promoted genomic changes. HEK293_*CHI3L1* and HeLa_*CHI3L1* cells demonstrated the opposite growth characteristics [3]. The long-term treatment with DNA-damaging drug temozolomide increased genomic diversity of U251 and T98G cells but selected genetically stable C6 clones [4]. The long-term treatment with mTOR inhibitor temsirolimus or MEK1/2 inhibitor U0126 increased genomic heterogeneity of U251 and T98G cells [5]. The long-term drug-treated cells demonstrated distinct, cell line- and treatment-dependent, phenotypic changes. An increase of resistance to drug re-challenge was the only predictable phenotypic trait intrinsic to all long-term drug-treated tumor cells [4,5].

**Conclusions**

A stable transfection of plasmid DNA into tumor cells can result in chromosomal abnormalities and phenotypic changes challenging an assumption that empty vector-transfected cells preserve the cytogenetic and phenotypic characteristics and represent the adequate control in transfection experiments [6]. The opposite growth characteristics of *CHI3L1*-transfected tumor cells suggest that the effects and functions of a (trans)gene can be opposite and versatile in cells with the different genetic networks, defined by the genome context [7]**.** The long-term drug treatment selects resistant genotype-phenotype variants or generates novel versatile phenotypes by increasing genomic chaos. Genomic instability-driven multilayered heterogeneity and complex reprogramming of signal transduction pathways are responsible for adaptation of a tumor cell population to cytotoxic stresses of different nature.

**References**

1. Stepanenko AA, Kavsan VM. Evolutionary karyotypic theory of cancer versus conventional cancer gene mutation theory. Biopolym Cell 2012;28:267–80. doi:10.7124/bc.000059.

2. Stepanenko AA, Kavsan VM. Immortalization and malignant transformation of Eukaryotic cells. Cytol Genet 2012;46:96–129. doi:10.3103/S0095452712020041.

3. Stepanenko A, Andreieva S, Korets K, Mykytenko D, Huleyuk N, Vassetzky Y, et al. Step-wise and punctuated genome evolution drive phenotype changes of tumor cells. Mutat Res Mol Mech Mutagen 2015;771:56–69. doi:10.1016/j.mrfmmm.2014.12.006.

4. Stepanenko AA, Andreieva S V, Korets K V, Mykytenko DO, Baklaushev VP, Huleyuk NL, et al. Temozolomide promotes genomic and phenotypic changes in glioblastoma cells. Cancer Cell Int 2016;16:36. doi:10.1186/s12935-016-0311-8.

5. Stepanenko AA, Andreieva SV, Korets KV, Mykytenko DO, Baklaushev VP, Chekhonin VP, et al. mTOR inhibitor temsirolimus and MEK1/2 inhibitor U0126 promote chromosomal instability and cell type-dependent phenotype changes of glioblastoma cells. Gene 2016;579:58–68. doi:10.1016/j.gene.2015.12.064.

6. Stepanenko AA, Dmitrenko V V. HEK293 in cell biology and cancer research: phenotype, karyotype, tumorigenicity, and stress-induced genome-phenotype evolution. Gene 2015;569:182–90. doi:10.1016/j.gene.2015.05.065.

7. Stepanenko AA, Vassetzky YS, Kavsan VM. Antagonistic functional duality of cancer genes. Gene 2013;529:199–207. doi:10.1016/j.gene.2013.07.047.

## A10 Assessing chromosome copy number heterogeneity in cancer and other ageing-associated disease

### Bjorn Bakker^1^, Aaron S. Taudt^1^, Mirjam E. Belderbos^1,3^, David Porubsky^1^, Diana C.J. Spierings^1^, Tristan V. de Jong^1^, Nancy Halsema^1^, Hinke G. Kazemier^1^, Karina Hoekstra-Wakker^1^, Allan Bradley^2^, Eveline S.J.M. de Bont^3^, Anke van den Berg^4^, Victor Guryev^1^, Peter M. Lansdorp^1,5,6^, Maria Colomé Tatché^1,5,7^, Floris Foijer^1^

#### ^1^European Research Institute for the Biology of Ageing, University of Groningen, University Medical Center Groningen, A. Deusinglaan 1, NL-9713 AV Groningen, The Netherlands; ^2^Wellcome Trust Sanger Institute, Wellcome Trust Genome Campus, Hinxton, CB10 1SA, UK; ^3^Department of Paediatrics, University of Groningen, University Medical Center Groningen, A. Deusinglaan 1, NL-9713 AV Groningen, The Netherlands; ^4^Department of Pathology & Medical Biology, University of Groningen, University Medical Center Groningen, A. Deusinglaan 1, NL-9713 AV Groningen, The Netherlands; ^5^Terry Fox Laboratory, BC Cancer Agency, Vancouver, BC V5Z 1 L3, Canada; ^6^Department of Medical Genetics, University of British Columbia, Vancouver, BC, V6T 1Z4, Canada; ^7^Institute for Computational Biology, Helmholtz Zentrum München, Ingolstädter Landstr. 1, 85764, Neuherberg, Germany

##### **Correspondence:** Floris Foijer (f.foijer@umcg.nl)

Two out of three tumors display an abnormal chromosome content, a state defined as aneuploid. Aneuploidy is the result of cells missegregating chromosomes during mitosis, also known as chromosomal instability (CIN). Despite being a hallmark feature of cancer cells, aneuploidy inhibits cell proliferation of non-transformed cells, suggesting that cancer cells have acquired mutations that help them cope with the disadvantages of aneuploidy. This makes aneuploidy a promising target for cancer therapy [1]. To better understand how cells cope with aneuploidy *in vivo*, we developed conditional knockout mouse models, in which we can provoke a high rate of constant chromosome missegregation events in tissues of choice. Our models have allowed us to induce highly aneuploid cancers in various tissues in a p53-deficient setting, including liver and T-cells in a highly reproducible fashion. Surprisingly, when we measured the average DNA content in a large number of these aneuploid tumors, we found that tumors exhibited recurrent copy number changes in each tumor for some chromosomes, but not others [2]. This suggested that either the tumors had overcome the mutations that provoked the constant chromosome missegregation or that the recurring copy number changes were outcompeting the missegregation rate. To test this, we performed single cell sequencing of primary tumor cells and developed AneuFinder, a dedicated software tool to quantify intratumour karyotype heterogeneity [3]. AneuFinder analysis of our murine aneuploid lymphoma revealed dramatic intratumour heterogeneity, while confirming selection for the recurrently gained/lost chromosomes, indicating ongoing chromosome missegregation can yield seemingly stable aneuploid tumors that display high-grade intratumour heterogeneity. We are currently analyzing human tumors and our preliminary findings are indicating that intratumour heterogeneity rates can be very different between aneuploid tumors. As ongoing CIN and the resulting heterogeneity might predispose tumors to become therapy resistant [4], unbiased single cell karyotype assessment might become an essential tool in the future to stratify cancer therapy.

**References**

1. Sheltzer JM, Amon A: The aneuploidy paradox: costs and benefits of an incorrect karyotype. *Trends Genet* 2011, 27:446–53.

2. Foijer F, Xie SZ, Simon JE, Bakker PL, Conte N, Davis SH, Kregel E, Jonkers J, Bradley A., Sorger PK: Chromosome instability induced by Mps1 and p53 mutation generates aggressive lymphomas exhibiting aneuploidy-induced stress. *Proc Natl Acad Sci* 2014.

3. Bakker B, Taudt A, Belderbos M, Porubsky D, Spierings D, De T, Halsema N, Kazemier I, Hoekstra-wakker K, Bradley A, Bont E De, Haan G De, Guryev V, Lansdorp PM, Tatché MC, Foijer F: Single cell sequencing reveals karyotype heterogeneity in murine and human tumours. *Genome Biol* 2016, 17:1–15.

4. Sotillo R, Schvartzman J-M, Socci ND, Benezra R: Mad2-induced chromosome instability leads to lung tumour relapse after oncogene withdrawal. *Nature* 2010, 464:436–40.

## A11 What about benign chromosomal imbalances in cancer genetics

### Thomas Liehr (Thomas.Liehr@med.uni-jena.de)

#### Jena University Hospital, Friedrich Schiller University, Institute of Human Genetics, Jena, Germany

Copy number variations (CNVs) comprise microscopically visible harmless CNVs (CG-CNVs), known as heteromorphisms, as well as submicroscopic CNVs in the size of kilo- to megabasepairs (MG-CNVs). While CG-CNVs are known since decades, MG-CNVs were discovered as major part of human genome in 2004. CG-CNVs as well as MG-CNVs can be found in clinically healthy individuals as well as cancer patients [1]. Here I provide a review not given/published before, on what is known today on the still too little studied harmless human CG-CNVs and MG-CNVs and their implications for cancer research and diagnostics.

Due to technical issues, only in CG-CNVs heterochromatic and euchromatic variants can be distinguished, while in MG-CNVs exclusively euchromatic variants can be detected. Heterochromatic CG-CNVs already lead in leukemia cases to false positive ‘detection’ of monosomies (like -7), due to a “cen-“ heteromorphis in one homologous chromosome. Also inversion heteromorphisms of chromosome 9, or less known euchromatic variants of 8pter or 16p11.2, can be confused with associated malignancy and meaningful acquired aberrations. Maybe due to the sheer amount of them it is yet completely unclear and not studied, if MG-CNVs may be associated with tumor subtypes, or not.

In summary, CG-CNVs and MG-CNVs are considered not at all, or at least not enough, yet during evaluation and reporting; this is true for constitutional as well as cancer genetics. Remembering the so-called two-hit model, suggesting that combination of per se harmless CNVs may lead to clinical aberrations if they are present together in one patient, makes the difficulties we are facing clearly evident, when thinking about which genetic changes are relevant for a disease and which are not.

**References**

1. Liehr T. Benign & Pathological Chromosomal Imbalances. Microscopic and Submicroscopic Copy Number Variations (CNVs) in Genetics and Counseling. 1st edition. New York. Academic Press; 2014.

## A12 The fate of newly formed tetraploid cells: evolution of centrosome and chromosome number

### Nicolaas C. Baudoin^1^, Joshua M. Nicholson^1^, Kimberly Soto^1^, Isabel Quintanilla^2^, Jordi Camps^2^, Daniela Cimini^1^

#### ^1^Department of Biological Sciences and Biocomplexity Institute, Virginia Tech, Blacksburg, VA, 24061, USA; ^2^Gastrointestinal and Pancreatic Oncology Group, Institut d'Investigacions Biomèdiques August Pi i Sunyer (IDIBAPS), Hospital Clínic de Barcelona, 08036, Barcelona, Spain

##### **Correspondence:** Daniela Cimini (cimini@vt.edu)

**Background**

Many lines of evidence have led to a widely accepted model for how tetraploidy may act as a precursor of chromosomal instability in cancer [1]. The model proposes that tetraploid cells, because of supernumerary centrosomes, will undergo highly defective chromosome segregation in mitosis. Such defective mitoses would generate cells with abnormal karyotypes and abnormal centrosome numbers, both of which are common in cancer cells. However, we [2] and others [3] have found that tetraploid cell clones with normal centrosome numbers can evolve after cytokinesis failure. The goal of our study was to identify the events that lead to the emergence of such clones, with the idea that this will help us understand how tetraploidy may be linked to the aneuploidy and extra centrosomes seen in cancer cells.

**Materials and Methods**

**Fig. 1 (abstract A12).**
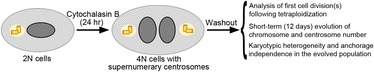
Diagrammatic representation of the experimental protocol used to generate tetraploid cell populations and list (right) of the analyses subsequently performed on such populations.

To achieve our goal, we examined the short-term evolution of experimentally-generated tetraploid cell populations. Such populations were generated by experimentally inhibiting cytokinesis in DLD1 cells (2 N) to obtain a population of prevalently tetraploid (4 N) cells, which also carried extra centrosomes (Fig. 1).

**Results**

We found that first generation tetraploid cells frequently, but not always, divide in a multipolar fashion. Moreover, chromosomes from multiple poles often cluster into a single daughter cell during cell division, but cell death and cell cycle arrest occur at high rates immediately after tetraploidization. By performing chromosome counts in the evolving population at regular time intervals, we found that highly aneuploidy karyotypes (between 2 N and 4 N) are generated as a result of multipolar divisions in tetraploid cells. However, such highly aneuploid karyotypes are quickly eliminated from the proliferating population and at day 12, two subpopulations remain with modal chromosome numbers of 46 and 90-92. Preliminary modeling efforts suggest that multipolar divisions would frequently produce cells with nullisomy, which may explain the reduced viability of cells produced by multipolar divisions. We also found that the fraction of cells possessing >2 centrosomes quickly decreases to very low levels during the 12-day period, despite a large fraction of the cell population still displaying near-tetraploid karyotypes at day 12. Finally, we determined that the evolved population displays high chromosome number heterogeneity and can form larger colonies in soft agar compared to the parental (2 N) cell population.

**Conclusions**

Overall, our data indicate that extra centrosomes are quickly lost during initial evolution of tetraploid cell populations. However, the remaining population displays high karyotypic heterogeneity, indicating that this, rather than the abnormal centrosome number may explain the tumorigenic potential of tetraploidy.

**References**

1. Storchova Z, Pellman D: From polyploidy to aneuploidy, genome instability and cancer. *Nat Rev Mol Cell Biol* 2004, 5(1):45-54.

2. Nicholson JM, Johnson GC, Cimini D: The effect of tetraploidy on chromosome mis-segregation and aneuploidy. *Mol Biol Cell* 2013, 24(24):2510.

3. Godinho SA, Picone R, Burute M, Dagher R, Su Y, Leung CT, Polyak K, Brugge JS, Thery M, Pellman D: Oncogene-like induction of cellular invasion from centrosome amplification. *Nature* 2014, 510(7503):167-171.

## A13 A systems biology perspective on the consequences of aneuploidy in human cells

### M. Dürrbaum^1,2^, N. Donnelly^1^, V. Passerini^1^, C. Kruse^1^, B. Habermann^2^, Z. Storchová^1,3^

#### ^1^Group Maintenance of Genome Stability, Max Planck Institute of Biochemistry, Martinsried, Germany; ^2^Group Computational Biology, Max Planck Institute of Biochemistry, Martinsried, Germany; ^3^Department of Molecular Genetics, Technical University Kaiserslautern, Kaiserslautern, Germany

##### **Correspondence:** M. Dürrbaum (duerrbau@biochem.mpg.de)

Aneuploidy, an unbalanced number of chromosomes, severely affects cell physiology and is widespread in pathologies such as cancer. In model systems, whole-chromosome aneuploidy results in impaired proliferation, replication stress, disturbed proteostasis, and specific changes to the transcriptome and proteome [1-10]. Yet, the molecular mechanisms underlying these characteristic changes are not well understood.

To elucidate common molecular mechanisms, we analyzed the consequences of aneuploidy in human cells from a systems biology perspective. To this end, we acquired genome, transcriptome, proteome and microRNAome data from a series of previously established human model aneuploid cell lines with one or two extra chromosomes [11]. Our large-scale comparison of the aneuploid transcriptomes revealed that the gene expression changes affect common cellular pathways independently of the cell line, type, and origin of aneuploidy [12]. We found that the conserved pathway deregulations are largely similar to the transcriptome changes after autophagy inhibition or in heat shock transcription factor- (HSF1) deficient cells. Moreover, the aneuploid proteome closely resembles the proteome after HSP90 inhibition [4]. This is in good agreement with our findings that aneuploid cells suffer from proteotoxic stress characterized by impaired HSF1 activation, HSP90 protein folding deficiency, and downregulation of HSP90 client proteins.

We hypothesized that microRNAs may play an additional role in the complex response to aneuploidy. Small RNA sequencing data analysis revealed that the microRNAome is strongly altered in aneuploid cells. Moreover, integrated analysis of microRNAome, transcriptome and proteome data suggests that the deregulated microRNAs negatively affect the development, growth and proliferation of aneuploid cells. Additionally, we identified the microRNA hsa-miR-10a-5p to be commonly upregulated in the analyzed cell lines. Subsequent experiments suggested that hsa-miR-10a-5p protects aneuploid cells from starvation-induced shutdown of translation of ribosomal proteins.

Taken together, this systems biology perspective demonstrates that the common phenotypic response to aneuploidy is reflected in conserved changes to the microRNAome, transcriptome, and proteome. Moreover, our results indicate that disturbed proteostasis significantly shapes the response to aneuploidy and its effects are manifested in both transcriptome and proteome. In addition, we show that the deregulated microRNAome contributes to the response to aneuploidy by negatively affecting the growth of aneuploid cells on one hand and positively regulating the resistance to stress on the other hand. Our “omics” approach not only facilitates an understanding of the common consequences of aneuploidy by comparison of many different aneuploid cell lines, but also points to new avenues of research into aneuploidy in cancer.

**References**

1. Torres, E. M. et al. Effects of aneuploidy on cellular physiology and cell division in haploid yeast. *Science* 317, 916–924 (2007).

2. Stingele, S., Stoehr, G. & Storchova, Z. Activation of autophagy in cells with abnormal karyotype. *Autophagy* 9, 246–248 (2013).

3. Oromendia, A. B., Dodgson, S. E. & Amon, A. Aneuploidy causes proteotoxic stress in yeast. *Genes & Development* 26, 2696–2708 (2012).

4. Donnelly, N., Passerini, V., Dürrbaum, M., Stingele, S. & Storchova, Z. HSF1 deficiency and impaired HSP90-dependent protein folding are hallmarks of aneuploid human cells. *The EMBO Journal* 33, 2374–2387 (2014).

5. Passerini, V. et al. The presence of extra chromosomes leads to genomic instability. *Nature Communications* 7, 10754 (2016).

6. Nawata, H. et al. Dysregulation of Gene Expression in the Artificial Human Trisomy Cells of Chromosome 8 Associated with Transformed Cell Phenotypes. *PLoS ONE* 6, e25319 (2011).

7. Ariyoshi, K. et al. Induction of genomic instability and activation of autophagy in artificial human aneuploid cells. *Mutat. Res*. 790, 19–30 (2016).

8. Niwa, O., Tange, Y. & Kurabayashi, A. Growth arrest and chromosome instability in aneuploid yeast. *Yeast* 23, 937–950 (2006).

9. Ohashi, A. et al. Aneuploidy generates proteotoxic stress and DNA damage concurrently with p53-mediated post-mitotic apoptosis in SAC-impaired cells. *Nature Communications* 6, 7668 (2015).

10. Pavelka, N. et al. Aneuploidy confers quantitative proteome changes and phenotypic variation in budding yeast. *Nature* 468, 321–325 (2010).

11. Stingele, S. et al. Global analysis of genome, transcriptome and proteome reveals the response to aneuploidy in human cells. *Molecular Systems Biology* 8, 1–12 (2012).

12. Dürrbaum, M. et al. Unique features of the transcriptional response to model aneuploidy in human cells. *BMC Genomics* 15, 1–14 (2014).

## A14 Aneuploidy: an evidence-based biomarker for environmental health

### Daniele Mandrioli^1^, Fiorella Belpoggi^1^, Ellen K Silbergeld^2^, Melissa J Perry^3^

#### ^1^Cesare Maltoni Cancer Research Center, Ramazzini Institute, 40010 Bentivoglio, Bologna, Italy; ^2^Department of Environmental Health Sciences, Johns Hopkins Bloomberg School of Public Health, 21205 Baltimore, MD, USA; ^3^Department of Environmental and Occupational Health, Milken Institute School of Public Health, The George Washington University, 20052 Washington DC, USA

##### **Correspondence:** Daniele Mandrioli (mandriolid@ramazzini.it)

**Background**

Aneuploidy, defined as structural and numerical aberrations of the chromosomes, continues to draw attention as an informative effect biomarker for carcinogens and reproductive toxicants [1]. Aneuploidy is a hallmark of cancer and precancerous lesions, some of the most powerful carcinogens are capable of inducing aneuploidy in different tissues [2-6]. The higher the degree of aneuploidy in precancerous lesions, the higher the risk of malignant progression [7-16].

On the other hand, sperm aneuploidy represents both a biomarker of reproductive toxicity, associated with infertility, pregnancy loss and a number of congenital abnormalities [17], and also a biomarker of neoplastic and preneoplastic lesion for testicular cancer [18, 19]. In fact aneuploid cells from germinal neoplasias are present in the sperm of patients with testicular cancer. At least 7 substances are currently known to increase sperm aneuploidy [20]: phthalates [21], styrene [22], organophosphates [23], carbaryl [24], fenvalerate [25], lead [26, 27], and benzene [28]. Six of these 7 substances are also known or suspected carcinogens [29]. Exposures to DDT and PCBs, substances already classified as carcinogens, have been also recently associated with increased rates of sperm aneuploidy in human [17]. We are currently investigating sperm aneuploidy in association with the exposure to different pesticides, including glyphosate and pyrethroids, and chemicals, in particular per- and polyfluoroalkyl substances (PFAS), studying both humans cohorts and experimental animal models.

**Materials and Methods**

The animal model will be Sprague Dawley rats from the colony of the Cesare Maltoni Cancer Research Center, Ramazzini Institute. This model has been used for over 40 years for toxicology and carcinogenicity bioassays. Sperm obtained from Sprague Dawley rats will be analyzed through mFISH laser-scanning microscopy by the George Washington University, following similar procedures already in use for humans [17].

**Results**

As we recently pointed out [1], the association between sperm aneuploidy and chemical exposures has been scarcely tested in toxicological and epidemiological studies. Our recent results highlight the value of this biomarker through observational studies in humans exposed to pesticides and other chemicals [17]. Our preliminary results seem to indicate that mFISH, in combination with laser scanning microscopy, is a suitable technique for sperm aneuploidy analysis in Sprague Dawley rats exposed to chemicals.

**Conclusions**

Aneuploidy is a biomarker that may indicate exposures to substance that act as carcinogens and reproductive toxicants [1]. With the advent of automated chromosome counters and Laser Scanning Microscopy [30], sperm aneuploidy assessment has become much faster and reliable, in both human and experimental models. Therefore new toxicological and epidemiological studies, using state-of the art mFISH techniques, are necessary to evaluate the burden of chemicals that are capable of inducing sperm aneuploidy. Specific attention should be given to pesticides because of their widespread use and their frequent association with sperm aneuploidy, reproductive toxicity and carcinogenicity.

**Acknowledgements**

We authors would like to thank: the staff of the Cesare Maltoni Cancer Research Center, Ramazzini Institute; Linda Nguyen and Laura Neumann, George Washington University; Peter Duesberg and Amanda McCormack, UC Berkeley; Giovanni Pierini, University of Bologna; Francesco Ceci and Paolo Castellucci, Department of Nuclear Medicine, S.Orsola-Malpighi Hospital, Bologna; Tiziana Balbi, Institute of Surgical Pathology, S. Orsola-Malpighi Hospital.

**References**

1. Mandrioli D, Belpoggi F, Silbergeld EK, Perry MJ. Aneuploidy: a common and early evidence-based biomarker for carcinogens and reproductive toxicants. Environmental health : a global access science source. 2016 Oct 12;15(1):97. PubMed PMID: 27729050. Pubmed Central PMCID: PMC5059969. Epub 2016/10/13. eng.

2. Quick EL, Parry EM, Parry JM. Do oestrogens induce chromosome specific aneuploidy in vitro, similar to the pattern of aneuploidy seen in breast cancer? Mutation research. 2008 Mar 12;651(1-2):46-55. PubMed PMID: 18162433. Epub 2007/12/29. eng.

3. Storchova Z. The causes and consequences of aneuploidy in eukaryotic cells. Aneuploidy in health and disease Rijecka (Croatia): InTech Europe. 2012:1-21.

4. Duesberg P, Rasnick D, Li R, Winters L, Rausch C, Hehlmann R. How aneuploidy may cause cancer and genetic instability. Anticancer research. 1999 Nov-Dec;19(6a):4887-906. PubMed PMID: 10697602. Epub 2000/03/04. eng.

5. Tsutsui T, Maizumi H, McLachlan JA, Barrett JC. Aneuploidy induction and cell transformation by diethylstilbestrol: a possible chromosomal mechanism in carcinogenesis. Cancer research. 1983 Aug;43(8):3814-21. PubMed PMID: 6861146. Epub 1983/08/01. eng.

6. Tiwari D, Kamble J, Chilgunde S, Patil P, Maru G, Kawle D, et al. Clastogenic and mutagenic effects of bisphenol A: an endocrine disruptor. Mutation research. 2012 Mar 18;743(1-2):83-90. PubMed PMID: 22245107. Epub 2012/01/17. eng.

7. Elzagheid A, Kuopio T, Collan Y. Implementation of DNA cytometric measurements in fine-needle aspiration biopsy diagnostics of breast disease. Cancer. 2004 Dec 25;102(6):380-8. PubMed PMID: 15494977. Epub 2004/10/21. eng.

8. Steinbeck RG. Proliferation and DNA aneuploidy in mild dysplasia imply early steps of cervical carcinogenesis. Acta oncologica (Stockholm, Sweden). 1997;36(1):3-12. PubMed PMID: 9090961. Epub 1997/01/01. eng.

9. Li G, Guillaud M, LeRiche J, McWilliams A, Gazdar A, Lam S, et al. Automated sputum cytometry for detection of intraepithelial neoplasias in the lung. Analytical cellular pathology (Amsterdam). 2012;35(3):187-201. PubMed PMID: 22277916. Pubmed Central PMCID: Pmc3412676. Epub 2012/01/27. eng.

10. Massion PP, Zou Y, Uner H, Kiatsimkul P, Wolf HJ, Baron AE, et al. Recurrent genomic gains in preinvasive lesions as a biomarker of risk for lung cancer. PloS one. 2009;4(6):e5611. PubMed PMID: 19547694. Pubmed Central PMCID: Pmc2699220. Epub 2009/06/24. eng.

11. Baretton G, Vogt T, Valina C, Schneiderbanger K, Lohrs U. [Prostate cancers and potential precancerous conditions: DNA cytometric investigations and interphase cytogenetics]. Verhandlungen der Deutschen Gesellschaft fur Pathologie. 1993;77:86-92. PubMed PMID: 7511309. Epub 1993/01/01. Prostata-Karzinom und potentielle Prakanzerosen: DNA-zytometrische Untersuchungen und Interphasenzytogenetik. ger.

12. North JP, Garrido MC, Kolaitis NA, LeBoit PE, McCalmont TH, Bastian BC. Fluorescence in situ hybridization as an ancillary tool in the diagnosis of ambiguous melanocytic neoplasms: a review of 804 cases. The American journal of surgical pathology. 2014 Jun;38(6):824-31. PubMed PMID: 24618603. Epub 2014/03/13. eng.

13. Giaretti W, Monteghirfo S, Pentenero M, Gandolfo S, Malacarne D, Castagnola P. Chromosomal instability, DNA index, dysplasia, and subsite in oral premalignancy as intermediate endpoints of risk of cancer. Cancer epidemiology, biomarkers & prevention : a publication of the American Association for Cancer Research, cosponsored by the American Society of Preventive Oncology. 2013 Jun;22(6):1133-41. PubMed PMID: 23629518. Epub 2013/05/01. eng.

14. Menke-Pluymers MB, Mulder AH, Hop WC, van Blankenstein M, Tilanus HW. Dysplasia and aneuploidy as markers of malignant degeneration in Barrett's oesophagus. The Rotterdam Oesophageal Tumour Study Group. Gut. 1994 Oct;35(10):1348-51. PubMed PMID: 7959183. Pubmed Central PMCID: Pmc1375001. Epub 1994/10/01. eng.

15. Singh M, Mehrotra S, Kalra N, Singh U, Shukla Y. Correlation of DNA ploidy with progression of cervical cancer. Journal of cancer epidemiology. 2008;2008:298495. PubMed PMID: 20445775. Pubmed Central PMCID: Pmc2858932. Epub 2008/01/01. eng.

16. Haase D, Germing U, Schanz J, Pfeilstocker M, Nosslinger T, Hildebrandt B, et al. New insights into the prognostic impact of the karyotype in MDS and correlation with subtypes: evidence from a core dataset of 2124 patients. Blood. 2007 Dec 15;110(13):4385-95. PubMed PMID: 17726160. Epub 2007/08/30. eng.

17. Perry MJ, Young HA, Grandjean P, Halling J, Petersen MS, Martenies SE, et al. Sperm Aneuploidy in Faroese Men with Lifetime Exposure to Dichlorodiphenyldichloroethylene (p,p -DDE) and Polychlorinated Biphenyl (PCB) Pollutants. Environmental health perspectives. 2016 Jul;124(7):951-6. PubMed PMID: 26535963. Pubmed Central PMCID: PMC4937854. Epub 2015/11/05. eng.

18. Giwercman A, Clausen OP, Skakkebaek NE. Carcinoma in situ of the testis: aneuploid cells in semen. British medical journal (Clinical research ed). 1988 Jun 25;296(6639):1762-4. PubMed PMID: 3136829. Pubmed Central PMCID: Pmc2546236. Epub 1988/06/25. eng.

19. Clausen OP, Giwercman A, Jorgensen N, Bruun E, Frimodt-Moller C, Skakkebaek NE. DNA distributions in maldescended testes: hyperdiploid aneuploidy without evidence of germ cell neoplasia. Cytometry. 1991;12(1):77-81. PubMed PMID: 1671832. Epub 1991/01/01. eng.

20. Schrader SM, Marlow KL. Assessing the reproductive health of men with occupational exposures. Asian J Androl. 2014 Jan-Feb;16(1):23-30. PubMed PMID: 24369130. Pubmed Central PMCID: PMC3901877. Epub 2013/12/27. eng.

21. Huang LP, Lee CC, Hsu PC, Shih TS. The association between semen quality in workers and the concentration of di(2-ethylhexyl) phthalate in polyvinyl chloride pellet plant air. Fertil Steril. 2011 Jul;96(1):90-4. PubMed PMID: 21621774. Epub 2011/05/31. eng.

22. Migliore L, Naccarati A, Zanello A, Scarpato R, Bramanti L, Mariani M. Assessment of sperm DNA integrity in workers exposed to styrene. Human reproduction (Oxford, England). 2002 Nov;17(11):2912-8. PubMed PMID: 12407048. Epub 2002/10/31. eng.

23. Sanchez-Pena LC, Reyes BE, Lopez-Carrillo L, Recio R, Moran-Martinez J, Cebrian ME, et al. Organophosphorous pesticide exposure alters sperm chromatin structure in Mexican agricultural workers. Toxicol Appl Pharmacol. 2004 Apr 1;196(1):108-13. PubMed PMID: 15050412. Epub 2004/03/31. eng.

24. Xia Y, Cheng S, Bian Q, Xu L, Collins MD, Chang HC, et al. Genotoxic effects on spermatozoa of carbaryl-exposed workers. Toxicological sciences: an official journal of the Society of Toxicology. 2005 May;85(1):615-23. PubMed PMID: 15615886. Epub 2004/12/24. eng.

25. Bian Q, Xu LC, Wang SL, Xia YK, Tan LF, Chen JF, et al. Study on the relation between occupational fenvalerate exposure and spermatozoa DNA damage of pesticide factory workers. Occupational and environmental medicine. 2004 Dec;61(12):999-1005. PubMed PMID: 15550606. Pubmed Central PMCID: Pmc1740696. Epub 2004/11/20. eng.

26. Naha N, Chowdhury AR. Inorganic lead exposure in battery and paint factory: effect on human sperm structure and functional activity. Journal of UOEH. 2006 Jun 1;28(2):157-71. PubMed PMID: 16780224. Epub 2006/06/20. eng.

27. Hsu PC, Chang HY, Guo YL, Liu YC, Shih TS. Effect of smoking on blood lead levels in workers and role of reactive oxygen species in lead-induced sperm chromatin DNA damage. Fertil Steril. 2009 Apr;91(4):1096-103. PubMed PMID: 18342860. Epub 2008/03/18. eng.

28. Marchetti F, Eskenazi B, Weldon RH, Li G, Zhang L, Rappaport SM, et al. Occupational exposure to benzene and chromosomal structural aberrations in the sperm of Chinese men. Environmental health perspectives. 2012 Feb;120(2):229-34. PubMed PMID: 22086566. Pubmed Central PMCID: Pmc3279447. Epub 2011/11/17. eng.

29. NTP. Report on Carcinogens (12th Ed.): DIANE Publishing Company; 2011.

30. Perry M. Chemically induced DNA damage and sperm and oocyte repair machinery: the story gets more interesting. Asian J Androl. 2015 May 19. PubMed PMID: 25994653. Epub 2015/05/23. Eng.

## A15 Transcriptome instability in cancer; Aberrant RNA processing, variants and chimeras

### Rolf I. Skotheim^1,2^, Marthe Løvf^1,2^, Bjarne Johannessen^1,2^, Andreas M. Hoff^1,2^, Sen Zhao^1,2^, Jonas M. SveeStrømme^1,2^, Anita Sveen^1,2^, Ragnhild A. Lothe^1,2^

#### ^1^Department of Molecular Oncology, Institute for Cancer Research, Oslo University Hospital, Oslo, Norway; ^2^Centre of Cancer Biomedicine, Medical Faculty, University of Oslo, Oslo, Norway

##### **Correspondence:** Rolf I. Skotheim (rolf.i.skotheim@rr-research.no)

We have described transcriptome instability (TIN) as a novel phenotype of colorectal cancer [1]. These findings were followed up by investigation of genome-scale and exon-level mRNA abundance levels from altogether 555 patients with primary cancers from seven different anatomical sites. We developed algorithms to score the sample-wise and transcriptome-wide amounts of alternative splicing. We found that TIN is a pan-cancer characteristic of carcinomas [2]. Interestingly, the amounts of aberrant exon skipping and inclusion in tumors, correlate significantly with expression levels of splicing factor genes. This was the case in cancers of the breast, cervix, colorectum, lung and prostate. Thus, we see a plausible biological explanation for the observed splicing variation.

Further, through whole-transcriptome sequencing, we and others see that there are large amounts of chimeric RNA molecules which are expressed both in malignant and non-malignant tissues [3-5]. These are commonly produced by mis-splicing (after RNA polymerase read through of adjacent genes or through trans-splicing of distantly located genes). Many of the chimeric RNAs are ectopically expressed in cancer samples, although not yet linked to the TIN phenotype. For example, the fusion transcript *SLC45A3-ELK4* is ubiquitously expressed in both normal and malignant tissues from prostate, but to a much higher degree in a subset of cancer samples [6-7; and unpublished]. To speed-up discovery of fusion transcripts, we developed a protocol for highly multiplexed rapid amplification of cDNA ends (RACE) coupled by high-throughput sequencing of pooled RACE-products from many patients (RACE-seq). We have used this RACE-seq approach to identify novel fusion transcripts from colorectal cancers [8].

**Conclusion**

We see an enormous variation in the RNA-transcript processing across cancer samples. This is commonly due to misregulation of the pre-mRNA splicing machinery, which also may generate chimeric RNA molecules.

**References**

1. Sveen A, Ågesen TH, Nesbakken A, Rognum TO, Lothe RA, Skotheim RI. Transcriptome instability in colorectal cancer identified by exon microarray analyses: Associations with splicing factor expression levels and patient survival. Genome Med. 2011; 3: 32

2. Sveen A, Johannessen B, Teixeira MR, Lothe RA, Skotheim RI. Transcriptome instability as a molecular pan-cancer characteristic of carcinomas. BMC Genomics. 2014; 15: 672

3. Nome T, Hoff AM, Bakken AC, Rognum TO, Nesbakken A, Skotheim RI. High frequency of fusion transcripts involving TCF7L2 in colorectal cancers: Novel fusion partner and splice variants. PLoS ONE. 2014; 9: e91264

4. Nome T, Thomassen GOS, Bruun J, Ahlquist TC, Bakken AC, Hoff AM, Rognum T, Nesbakken A, Lorenz S, et al. Common fusion transcripts identified in colorectal cancer cell lines by high throughput RNA sequencing. Transl Oncol. 2013; 6: 546-553

5. Hoff AM, Alagaratnam S, Zhao S, Bruun J, Andrews PW, Lothe RA, Skotheim RI. Identification of novel fusion genes in testicular germ cell tumors. Cancer Res. 2016; 76: 108-116

6. Rickman DS, Pflueger D, Moss B, Vandoren VE, Chen CX, de la TA, Kuefer R, Tewari AK, Setlur SR, Demichelis F, Rubin MA. SLC45A3-ELK4 Is a Novel and Frequent Erythroblast Transformation-Specific Fusion Transcript in Prostate Cancer. Cancer Res. 2009;

7. Kannan K, Wang L, Wang J, Ittmann MM, Li W, Yen L. Recurrent chimeric RNAs enriched in human prostate cancer identified by deep sequencing. Proc Natl Acad Sci U S A. 2011; 108: 9172-9177

8. Hoff AM, Johannessen B, Alagaratnam S, Zhao S, Nome T, Løvf M, Bakken AC, Hektoen M, Sveen A, Lothe RA, Skotheim RI. Novel RNA-variants in colorectal cancers. Oncotarget. 2015; 6: 36587-36602

## A16 The relevance of cytogenetic aberrations in human leukemia

### R. Hehlmann, A. Voskanyan, A. Fabarius

#### Medizinische Fakultät Mannheim, Universität Heidelberg, Heidelberg, Germany

##### **Correspondence:** R. Hehlmann (Sekretariat.Hehlmann@medma.uni-heidelberg.de)

Cytogenetic aberrations have been associated with diagnosis and prognosis of common human leukemias with increasing frequency. The detection of the Philadelphia chromosome with the t(9;22) translocation in chronic myeloid leukemia (CML) was the first chromosome aberration regularly associated with a definite type of leukemia. As molecular correlate of the t(9;22)translocation the juxtaposition of the ABL oncogene of chromosome 9 and BCR sequences of chromosome 22 was identified which results in a BCR-ABL fusion transcript. Genetic instability induced by BCR-ABL is thought to cause additional chromosomal aberrations (ACA) that promote clonal evolution, drug resistance and disease progression [1] pointing to a cooperation between the ABL-oncogene and aneuploidy.

Cytogenetic aberrations have been identified as diagnostic and prognostic markers also in chronic lymphocytic leukemia (CLL), acute myeloid leukemia (AML) and acute lymphoblastic leukemia (ALL): A new prognostic score has been proposed for CLL that uses cytogenetic aberrations to guide personalized therapy [2]. The 2016 revision of the World Health Organization classification of myeloid neoplasm and acute leukemias includes cytogenetic and molecular data for diagnostic and prognostic classification [3]. The new recommendations of the European LeukemiaNet for the management of AML [4] are based on cytogenetics and mutations for stratification of AML according to prognosis and targets for therapy.

Not all additional cytogenetic aberrations are prognostically equally relevant. In CML, only “classic “ major route aberrations [+8, +Ph, i(17)(q10),+19] at diagnosis have been associated with a poor prognosis [5], whereas minor route aberrations (balanced or unbalanced) have no negative impact [6]. In an analysis of ACA appearing during the course of CML more types of ACA (particularly -7 and 3q26) were identified that were associated with inferior survival [7]. ACA newly arising under therapy were defined as indicating clonal evolution and a poor prognosis by ELN management recommendations for CML [8].

In a correlation of ACA with blast counts it was determined that ACA, in particular major route ACA, but also abnormalities of chromosomes 3,7,21 and 17 precede blast increase. The impact of minor route ACA could be eliminated by imatinib indicating that minor route ACA may not be relevant for blastic progression and may rather be bystanders than drivers [9].

In summary, chromosomal aberrations may determine prognosis and survival in virtually all types of leukemia.

**References**

1. Koptyra M, Falinski R, Nowicki MO, Stoklosa T, Majsterek I, Nieborowska-Skorska M, et al. BCR/ABL kinase induces self-mutagenesis via reactive oxygen species to encode imatinib resistance. *Blood* 2006;108:319-327.

2. Pflug N, Bahlo J, Shanafelt TD, Eichhorst BF, Bergmann MA, Elter T, et al. Development of a comprehensive prognostic index for patients with chronic lymphocytic leukemia. *Blood* 2014;124:49-62.

3. Arber DA, Orazi A, Hasserjian R, Thiele J, Borowitz MJ, Le Beau MM, et al. The 2016 revision to the World Health Organization classification of myeloid neoplasms and acute leukemia. *Blood* 2016;127:2391-2405.

4. Döhner Hartmut ea. Diagnosis and Management of AML in Adults: 2017 ELN Recommendations from an International Expert Panel. *Blood* 2017;in press Blood 2016/733196.

5. Fabarius A, Leitner A, Hochhaus A, Muller MC, Hanfstein B, Haferlach C, et al. Impact of additional cytogenetic aberrations at diagnosis on prognosis of CML: long-term observation of 1151 patients from the randomized CML Study IV. *Blood* 2011;118:6760-6768.

6. Fabarius A, Kalmanti L, Dietz CT, Lauseker M, Rinaldetti S, Haferlach C, et al. Impact of unbalanced minor route versus major route karyotypes at diagnosis on prognosis of CML. *Ann Hematol* 2015;94:2015-2024.

7. Wang W, Cortes JE, Tang G, Khoury JD, Wang S, Bueso-Ramos CE, et al. Risk stratification of chromosomal abnormalities in chronic myelogenous leukemia in the era of tyrosine kinase inhibitor therapy. *Blood* 2016;127:2742-2750.

8. Baccarani M, Deininger MW, Rosti G, Hochhaus A, Soverini S, Apperley JF, et al. European LeukemiaNet recommendations for the management of chronic myeloid leukemia: 2013. *Blood* 2013;122:872-884.

9. Voskanyan A, Dietz CT, Fabarius AC, Lauseker M, Saußele S, Kalmanti L, et al. Major route additional chromosomal aberrations (ACA) precede increase of blasts in chronic myeloid leukemia (CML) independent of therapy. An analysis of CML studies III, IIIA and IV. *Jahrestagung der Deutschen, Österreichischen und Schweizerischen Gesellschaften für Hämatologie und Medizinische Onkologie* 2016:Abstract P201.

## A17 Grading the malignancy of prostate cancer by quantifying DNA-aneuploidy

### Alfred Böcking^1^, Stefan Biesterfeld^2^, Leonid Berynskyy^3^, Christof Börgermann^4^, Rainer Engers^5^, Josef Dietz^6^

#### ^1^Institute of Pathology City Hopsital of Düren, Düren, Gemany; ^2^Institute of Pathology, Koblenz, Germany; ^3^Institute of Pathology, Department of Cytopathology, University of Düsseldorf, Düsseldorf, Germany; ^4^City Hospital Düren, Department of Urology, Düren, Germany; ^5^Institute of Pathology, Lukas-Hospital, Neuss, Germany; ^6^Bundesverband Prostatakrebs Selbsthilfe, BPS, Bretten, Germany

##### **Correspondence:** Alfred Böcking (alfred.boecking@web.de)

**Background**

“Active surveillance” (AS) of low-grade, localized cancers of the prostate represents a conservative strategy that renounces aggressive therapy with unpleasant side effects. It is suitable for about 30% of newly diagnosed cases and depends on a low Gleason-score (GS) of 6 or 7a. As this subjective score is burdened by a low interobserver reproducibility of about 60%, >30% of AS-patients have to face a progress of their cancer within four years, that makes active therapy necessary. The degree of malignancy of most cancers depends on the degree of chromosomal aneuploidy. This can indirectly be quantified by automated measurements the DNA-content of thousands of cancer cells, called “DNA-Karyometry.”

**Material and Methods**

In a prospective level 1b-study on 80 untreated prostate cancer patients under AS we have compared the ability of the subjective Gleason-Score (locals and reference pathologist R.E.) and the DNA-Grade of Malignancy (DNA-MG) to predict a biochemical or clinical progress within 4,1 years. Cancer cells were obtained from residual biopsies by enzymatic cell separation. After Feulgen staining at least 300 up to 30.000 cancer-nuclei have automatically been measured by TV-image-analysis, applying digital nuclear classifiers using a computerized microscope (MotiCyte-auto, Motic, Xiamen, P.R. China). DNA-MGs 1-4 have been distinguished according to the proposal of the ESACP (2001). GS >6 and DNA-MG >1 had been taken as predictors of progression. Proven stages pT > =3 and PSA-doubling times < 36 months were taken as indicators of progression. Other definitions have also been analyzed.

**Results**

While interobserver reproducibility of GS was found to be 55%, that of DNA-grading amounted to 92,7%. Sensitivity, specificity, positive and negative predicive values for reference pathologists GSs were: 22,2%, 93,1% 33,3% and 79,4%, that for DNA-grading: 100%, 80,0%, 33,3% and 100%.

**Conclusion**

By applying automated DNA-karyometry on residual prostate cancer biopsies additional to the GS, progressions of prostate cancers can be predicted or excluded more accurately. Thus patients can rely more safely on the AS-strategy that avoids side effects and complications of most active therapies.

**Authors’ information**

Alfred Böcking: Institute of Cytopathology University of Düsseldorf, Germany til 2009.

Stefan Biesterfeld: Institute of Pathology, Department of Cytopathology, University of Düsseldorf, Germany til 2015.

**References**

Böcking A, Tils M, Schramm M, Dietz J, Biesterfeld S. DNA-cytometric grading of prostate cancer. Systematic review with descriptive data analysis. *Pathol Discov* 2014; 2(7): 1-20

Böcking A. Comparability of tumor-cytogenetics and -DNA-cytometry. *Mol Cytogen* 2015; 8:28

Böcking A, Biesterfeld S, Dietz J, Haroske G, Kriegsmann, Motherby H, Falk S. Objektive DNA-Malignitäts-Gradierung als Ergänzung zum Gleason-Score. *Frankfurter Konsens. Pathologe* 2015; 36:498-502

## A18 Alterations in chromosome territory organization across stages of breast cancer progression

### A. Fritz^1^, N. Sehgal^2^, J. Vecerova^3^, B. Stojkovicz^4^, H. Ding^4^, N. Page^1^, C. Tye^1^, S. Bhattacharya^5^, J. Xu^4^, G. Stein^1^, J. Stein^1^, R. Berezney^2^

#### ^1^College of Medicine, Biochemistry, University of Vermont, Burlington, VT, USA; ^2^School of Medicine and Biomedical Services, Dermatology, SUNY Buffalo, NY, USA; ^3^Department of Biological Sciences, SUNY Buffalo, NY, USA; ^4^University at Buffalo, Department of Computer Science and Engineering, SUNY Buffalo, NY, USA; ^5^Department of Mathematics and Computer Science, Fayetteville State University, Fayetteville, NC, USA

##### **Correspondence:** A. Fritz (Andrew.Fritz@med.uvm.edu)

Changes in nuclear structure have long been used as a major diagnostic tool to detect cancer [1]. Although it is known that nuclear architecture is altered in cancer, less is known about the concomitant changes in CT organization. There is growing evidence that chromosome territories (CT) have a probabilistic non-random arrangement within the cell nucleus of mammalian cells including radial positioning and preferred patterns of interchromosomal interactions that are cell-type specific [2-7]. While it is generally considered that the three-dimensional (3D) arrangement of genes within the CT has a connection to genomic regulation, the spatial arrangement of some genes are more influenced by expression than others [8]. We examined the interchromosomal spatial positioning of a subset of human chromosomes in the human breast cell line MCF10A (10A) and its malignant counterpart MCF10CA1a (CA1a) [9]. Each CT was in close proximity with a similar number of other CT except the inactive CTXi and the gene rich CT17. The inactive X had lower levels of interchromosomal partners in 10A which increased strikingly in CA1a. In contrast chr17 had higher levels of interchromosomal interactions. Major alterations from 10A to CA1a were detected in the pairwise interaction profiles which were subdivided into five types of altered interaction profiles. Interestingly, we identified a pattern of differential interchromosomal interactions for each homolog of a given chromosome. We also found differential patterns with regard to nucleoli [10]. Global data mining program termed the chromatic median calculated the most probable overall association network for the entire subset of CT. This interchromosomal network was drastically altered in CA1a. Future studies will include the impact of aneuploidy on higher order chromatin organization. While we have identified global increased levels of transcription on aneuploid chromosomes, this relationship is not absolute. We conclude that CT undergo multiple and preferred interactions with other CT in the cell nucleus and form preferred-albeit probable-interchromosomal networks. This network of interactions is highly altered in malignant human breast cells. Aberrations in the karyotype of cancer cells is also expected to alter these interchromosomal interactions. It is intriguing to consider the relationship of these alterations to the corresponding changes in the gene expression program of malignant cancer cells.

**References**

1. Zink D, Fischer AH, Nickerson JA. Nuclear structure in cancer cells. *Nature Reviews Cancer* 2004;4(9):677-687.

2. Cremer M, von Hase J, Volm T, Brero A, Kreth G, Walter J, Fischer C, Solovei I, Cremer C, Cremer T. Non-random radial higher-order chromatin arrangements in nuclei of diploid human cells. *Chromosome Res* 2001;9(7):541–567.

3. Bolzer A, Kreth G, Solovei I, Koehler D, Saracoglu K, Fauth C, Muller S, Eils R, Cremer C, Speicher MR, et al. Three-dimensional maps of all chromosomes in human male fibroblast nuclei and prometaphase rosettes. *PLoS Biol* 2005;3(5):e157.

4. Parada LA, Roix JJ, Misteli T. An uncertainty principle in chromosome positioning. *Trends Cell Biol* 2003;13(8):393–396.

5. Parada LA, McQueen PG, Misteli T. Tissue-specific spatial organization of genomes. *Genome Biol* 2004;5(7):R44.

6. Bickmore WA. The spatial organization of the human genome. *Annu Rev Genomics Hum Genet* 2013;14:67–84.

7. Fritz AJ, Stojkovic B, Ding H, Xu J, Bhattacharya S, Berezney R. Cell type specific alterations in interchromosomal networks across the cell cycle. *PLoS Comput. Biol* 2014;10(10): e1003857.

8. Karen J. Meaburn. Spatial Genome Organization and Its Emerging Role as a Potential Diagnosis Tool. *Front Genet* 2016;7:134.

9. Fritz AJ, Stojkovic B, Ding H, Xu J, Bhattacharya S, Gaile D, Berezney R. Wide-scale alterations in interchromosomal organization in breast cancer cells: defining a network of interacting chromosomes. *Hum Mol Genet* 2014;23(19):5133–5146.

10. Pliss A, Fritz AJ, Stojkovic B, Ding H, Mukherjee L, Bhattacharya S, Xu J, Berezney R. Non-Random Patterns in the Distribution of NOR-Bearing Chromosome Territories in *Human Fibroblasts: A Network Model of Interactions* 2014;230(2):427–439.

## A19 Acute myeloid leukemias with complex karyotype harbor cryptic gene fusions that are candidate leukemic drivers

### Xue Gong^1,2^, Sarah Grasedieck^1,2^, Julian Swoboda^1,2^, Frank G. Rücker^2^, Lars Bullinger^2^, Jonathan R. Pollack

#### ^1^Department of Pathology, Stanford University, Stanford, California, 94010, USA; ^2^Department of Internal Medicine III, University Hospital of Ulm, Ulm, 89081, Germany

##### **Correspondence:** Jonathan R. Pollack (pollack1@stanford.edu)

Approximately 10-15% of acute myeloid leukemia (AML) cases are categorized as “complex karyotype”, defined as having three or more chromosome aberrations in the absence of prognostically-favorable rearrangements (t(8;21), inv(16), and t(15;17)) [1]. Complex karyotype AML is associated with unfavorable prognosis, and molecularly-targeted therapies have been lacking. Complex karyotype AML cases harbor multiple chromosome abnormalities, in many instances 10 or more, including numerical gains and losses, as well as varied structural rearrangements. Chromosome gains and losses are thought to impact the expression levels of residing oncogenes and tumor suppressors [2, 3]. However, the contribution of chromosome structural rearrangements has been less clear.

To investigate the impact of chromosome aberrations in complex karyotype AML, we carried out whole-transcriptome RNA sequencing (RNAseq) of 65 cases. All cases were associated with detailed clinicopathological annotations, cytogenetics, and SNP/CGH array profiles. RNAseq reads were aligned to the genome, and candidate gene fusions identified as reads spanning two different genes, using ChimeriScan and TopHat-Fusion. Selected fusions were independently verified by Nanopore sequencing.

In all, RNAseq analysis uncovered 55 high-evidence gene fusions in 30 (46% of) complex-karyotype AML samples. Nearly all fusions were previously unreported in AML. No gene fusions were recurrent, though some genes recurred as 5’ or 3’ partners. About one-quarter of fusions contained a known AML gene (e.g. *RUNX1, KMT2A*) fused to a gene previously unreported in AML. The majority of fusions comprised partners where neither was previously associated with AML, though many had plausible leukemic roles or targetable domains (e.g. cell surface receptors, kinases, other enzymes). Mechanistic studies of novel gene fusions are ongoing to elucidate functional roles.

The chromosome aberrations that comprise complex karyotype AML appear not only to impact gene expression levels, but also to conceal cryptic gene fusions that may contribute to leukemogenesis.

**References**

1. Mrozek, K, Cytogenetic, molecular genetic, and clinical characteristics of acute myeloid leukemia with a complex karyotype. *Semin Oncol* 2008;35(4):65-77.

2. Pollack, JR, Sorlie T, Perou CM, Rees CA, Jeffrey SS, Lonning PE, Tibshirani R, Botstein D, Borresen-Dale AL, Brown PO. Microarray analysis reveals a major direct role of DNA copy number alteration in the transcriptional program of human breast tumors. *Proc Natl Acad Sci USA* 2002;99(20):12963-8.

3. Salari K, Tibshirani R, Pollack JR. DR-Integrator: a new analytic tool for integrating DNA copy number and gene expression data. *Bioinformatics* 2010;26(3):414-6.

## A20 Chromosomal instability and cancer cell stemness under induced replication stress and extreme telomere dysfunction

### Fani-Marlen Roumelioti, Maria Chiourea, Christina Raftopoulou, Sarantis Gagos

#### Center for Experimental Medicine and Translational Research, Biomedical Research Foundation of the Academy of Athens Greece (BRFAA), Soranou Efesiou 4, Athens 115 27, Greece

##### **Correspondence:** Sarantis Gagos (sgagos@bioacademy.gr)

Chromosomal instability (CIN) in neoplasia generates extensive intra-tumor genomic heterogeneity that shapes the multistep process of malignancy and burdens onco-therapeutics. Human tumors and immortalized cell lines utilizing the Alternative lengthening of telomeres exert high rates of ongoing telomere dysfunction [1-3]. In ALT cells, numerical chromosomal aberrations are very frequent, while structural rearrangements affect almost every single chromosome [1-3]. This challenging context provides excellent grounds to study CIN in a single cell basis. Many cancers are considered to be driven by cancer stem cells (CSCs) that may differentiate into a variety of cell types while maintaining the ability to self-renew. To identify putative CSCs in the ALT-pathway, we combined single cell analysis by M-FISH/SKY, with a-CGH and Immunocytochemistry, in a panel of human ALT cell lines, before and after exposure to ionizing radiation, topoisomerase inhibition, or DNA replication-stress. Exogenous genotoxic stress triggered increased rates of random structural and numerical chromosome aberrations, polyploidization, as well as elevated frequencies of cancer cells expressing the mesenchymal CSC marker CD133. Enrichment of CD133+ cells in culture, showed significant decrease at the frequencies of telomere dysfunction foci and in random structural CIN, whereas the rates of whole genome endoreduplication, or polyploidy reduction, were highly elevated. Upon induced DNA damage, molecular karyotyping revealed several novel clonal structural chromosomal rearrangements distinguishing the challenged from the control cells. However by aCGH, the insulted ALT genomes displayed a remarkable propensity to maintain the major genomic imbalances of the control cells suggesting a trend that preserves monoclonality. Interestingly, CIN was unequally distributed between co-dividing cells both in control and challenged cell cultures. The minority of mitotic cells that appeared resistant to structural CIN, were found to represent products of polyploidization reduction, and corresponded well to the percentages of CD133+ cells. Our results demonstrate a continuous process of ALT cancer genome homeostasis that relies on polyploidization and polyploidy reduction and may be related to genotoxic therapy resistance and cancer cell stemness.

**References**

1. Sakellariou D, Chiourea M, Raftopoulou C, Gagos S. Alternative Lengthening of Telomeres: recurrent cytogenetic aberrations and chromosome stability under extreme telomere dysfunction. *Neoplasia* 2013;15(11):1301-1313.

2. Christodoulidou A, Raftopoulou C, Chiourea M, Papaioannou GK, Hoshiyama H, Wright WE, Shay JW, Gagos S. The Roles of Telomerase in the Generation of Polyploidy during Neoplastic Cell Growth. *Neoplasia* 2013;15(2):156-168.

3. Gagos S, Chiourea M, Christodoulidou A, Apostolou E, Raftopoulou C, Deustch S, Jefford CE, Irminger-Finger I, Shay JW, Antonarakis SE. Pericentromeric instability and spontaneous emergence of human neoacrocentric and minute chromosomes in the alternative pathway of telomere lengthening. *Cancer Research* 2008;68:8146-8155.

## A21 What if specific variations of the normal copy numbers of chromosomes cause cancer - independent of mutations?

### Peter Duesberg^1^, Mat Bloomfield^1,2^

#### ^1^Department Molecular and Cell Biology UC Berkeley, Donner Lab, Berkeley, CA, USA; ^2^Department of Natural Sciences and Mathematics, Dominican University, San Rafael, CA, USA

##### **Correspondence:** Peter Duesberg (duesberg@berkeley.edu)

**First genetic cancer theory: Specific aneusomies cause cancer**

By 1914 cancers were known to carry abnormal numbers of chromosomes and Theodor Boveri had discovered that individual chromosomes carry specific subsets of the cellular genome. On this basis Boveri advanced the theory that losses of specific chromosomes with growth “inhibitory” functions and gains of specific chromosomes with growth “stimulatory” functions are the causes of cancer [1]. This theory set off a search for the predicted, abnormal ‘cancer-specific’ chromosomes.

But, contrary to Boveri’s theory, the chromosomal abnormalities of all cancers tested were individual: No two cancers shared the same aneusomies [2-5]. According to the most recent account of the National Cancer Institute’s *NCI-Mitelman database* all 66,872 cancers recorded by November 2016 had individual sets of chromosomes or karyotypes [6].

**No theory to explain the individuality of the karyotypes of all cancers**

Despite the abundance of evidence for individual karyotypes in individual cancers, there is no theory to explain the individuality of cancer karyotypes in current textbooks [3, 7]. In view of this endless variety of cancer karyotypes most cancer researchers abandoned Boveri’s theory in favor of the theory of causal mutations, and thus regarded the individual karyotypes of cancers as “epiphenomena” [3, 8-10] or “Folgeerscheinungen” [11] or “consequences” [7] of the “instability” of cancer karyotypes [12, 13]. This confirms Gunther Stent’s famous theory for experimental scientists: “The best results are useless, if they cannot be confirmed by theory!”

In view of this, *Is it possible that cancer researchers have overlooked an established model for the generation of new functions and phenotypes by altering the karyotypes?*

**Speciation theory of cancer**

There is, indeed, a classic biological mechanism of altering phenotypes via karyotypes in countless variations – namely speciation.

By realizing that autonomy, individual karyotypes, transcriptomes and phenotypes and low probability of occurrence compared to mutation [12, 14-18] are all shared by carcinogenesis and speciation – a small group of cancer researchers has recently studied carcinogenesis as a form of speciation. Their names and abstracts are listed in the proceedings of this conference and representatives of each group have spoken at this conference, namely Mark Vincent, Henry Heng, Aleksei Stepanenko and us.

*Mechanism of carcinogenesis by speciation*. According to the speciation theory carcinogens or spontaneous accidents initiate carcinogenesis by inducing random aneuploidy. Aneuploidy then catalyzes random karyotypic variations automatically by unbalancing thousands of genes. Because of the very low probability that random karyotypic variation would form the karyotype of a new autonomous species, the karyotypes of cancers would be individual and clonal – much like those of conventional species. This would predict the karyotypic individuality [6] and clonal origins of cancers [18, 19].

*Clonal karyotypes, the most critical prediction of speciation theory.* To test this critical prediction of the speciation theory - we have developed a technique in which we detect chromosomal clonality even if a fraction of the chromosomes of a karyotype is non-clonal – as is typical for cancers [20-22]. Accordingly, we compared the copy numbers of individual chromosomes of 20 cancer karyotypes in 3-dimensional tables, hence termed *karyotype arrays*. The numbers of individual chromosomes are on the x-axis, the copy numbers of the chromosomes on the y-axis, and the numbers of karyotypes analyzed on the z-axis [22, 23]. The resulting parallel (clonal) lines formed by the karyotype arrays of all cancers tested so far, including breast, colon, cervical, pancreatic, and liver cancers directly confirmed the speciation theory [18, 21-26].

In the meantime complete genome sequencing of cancer cells undermines the conventional mutation theory [7, 12, 14, 27-30] by an inflation of thousands of new cancer-specific, but untested mutations [31-33] – leaving the speciation theory as the only probable alternative.

**Conclusion**

We conclude that cancers are generated and maintained by individual clonal karyotypes and corresponding transcriptomes as a whole – much like conventional species. Thus cancers are species of their own.

**References**

1. Boveri T, Harris H: Concerning the origin of malignant tumours by Theodor Boveri. Translated and annotated by Henry Harris. *J Cell Sci* 2008, 121 (Supplement 1):1-84.

2. Winge O: Zytologische Untersuchungen ueber die Natur maligner Tumoren. II. Teerkarzinome bei Maeusen. *Zeitschrift fuer Zellforschung und Mikroskopische Anatomie* 1930, 10:683-735.

3. Heim S, Mitelman F: Cancer Cytogenetics, Second edn. New York: Wiley-Liss; 1995.

4. Sandberg AA: The chromosomes in human cancer and leukemia, Second edn. New York: Elsevier Science Publishing; 1990.

5. Davidson JM, Gorringe KL, Chin SF, Orsetti B, Besret C, Courtay-Cahen C, Roberts I, Theillet C, Caldas C, Edwards PA: Molecular cytogenetic analysis of breast cancer cell lines. *Br J Cancer* 2000, 83(10):1309-1317.

6. Mitelman F, Johansson B, Mertens F: NCI-Mitelman Database of Chromosome Aberrations and Gene Fusions in Cancer, http://cgap.nci.nih.gov/Chromosomes/Mitelman. In*.*; 2016.

7. Weinberg RA: The biology of cancer, Second edition. edn. New York, NY ; London: Garland Science; 2014.

8. McMichael H, Wagner JE, Nowell PC, Hungerford DA: Chromosome Studies of Virus-Induced Rabbit Papillomas and Derived Primary Carcinomas. *J Natl Cancer Inst* 1963, 31:1197-1215.

9. Nowell PC, Hungerford DA, Cole LJ: Chromosome Changes Following Irradiation in Mammals. *Ann N Y Acad Sci* 1964, 114:252-258.

10. Sandberg AA: The chromosomes and causation of human cancer and leukemia. *Cancer Res* 1966, 26(9):2064-2081.

11. Andres AH: Zellstudien an Menschenkrebs. Der Chromosomale Bestand im Primaertumor und in der Metastase. *Z Zellforsch Mikrosk Anat* 1932, 16:88–122.

12. Alberts B, Johnson A, Lewis J, Raff M, Roberts K, Walter P: Molecular Biology of the Cell. New York: Garland Publishing, Inc.; 2014.

13. Heppner GH: Tumor heterogeneity. *Cancer Res* 1984, 44(6):2259-2265.

14. Nordling CO: A new theory on cancer-inducing mechanism. *Br J Cancer* 1953, 7(1):68-72.

15. Huxley J: Cancer biology: comparative and genetic. *Biol Rev* 1956, 31:474-514.

16. Pollack JR, Sorlie T, Perou CM, Rees CA, Jeffrey SS, Lonning PE, Tibshirani R, Botstein D, Borresen-Dale AL, Brown PO: Microarray analysis reveals a major direct role of DNA copy number alteration in the transcriptional program of human breast tumors. *Proc Natl Acad Sci U S A* 2002, 99(20):12963-12968.

17. Landry JJ, Pyl PT, Rausch T, Zichner T, Tekkedil MM, Stutz AM, Jauch A, Aiyar RS, Pau G, Delhomme N *et al*: The genomic and transcriptomic landscape of a HeLa cell line. *G3 (Bethesda)* 2013, 3(8):1213-1224.

18. Duesberg P, Mandrioli D, McCormack A, Nicholson JM: Is carcinogenesis a form of speciation? *Cell Cycle* 2011, 10(13):2100-2114.

19. McCormack A, Fan JL, Duesberg M, Bloomfield M, Fiala C, Duesberg P: Individual karyotypes at the origins of cervical carcinomas. *Mol Cytogenet* 2013, 6(1):44.

20. Duesberg P, Rausch C, Rasnick D, Hehlmann R: Genetic instability of cancer cells is proportional to their degree of aneuploidy. *Proc Natl Acad Sci U S A* 1998, 95(23):13692-13697.

21. Li L, McCormack AA, Nicholson JM, Fabarius A, Hehlmann R, Sachs RK, Duesberg PH: Cancer-causing karyotypes: chromosomal equilibria between destabilizing aneuploidy and stabilizing selection for oncogenic function. *Cancer Genet Cytogenet* 2009, 188(1):1-25.

22. Bloomfield M, Duesberg P: Karyotype alteration generates the neoplastic phenotypes of SV40-infected human and rodent cells. *Mol Cytogenet* 2015, 8:79.

23. Bloomfield M, Duesberg P: Inherent variability of cancer-specific aneuploidy generates metastases. *Mol Cytogenet* 2016, 9:90.

24. Nicholson JM, Duesberg P: On the karyotypic origin and evolution of cancer cells. *Cancer Genet Cytogenet* 2009, 194(2):96-110.

25. Stepanenko AA, Kavsan VM: Karyotypically distinct U251, U373, and SNB19 glioma cell lines are of the same origin but have different drug treatment sensitivities. *Gene* 2014, 540(2):263-265.

26. Bloomfield M, McCormack A, Mandrioli D, Fiala C, Aldaz CM, Duesberg P: Karyotypic evolutions of cancer species in rats during the long latent periods after injection of nitrosourea. *Mol Cytogenet* 2014, 7(1):71.

27. Cairns J: Cancer: Science and Society. San Francisco: W. H. Freeman; 1978.

28. Vogelstein B, Kinzler KW: The multistep nature of cancer. *Trends Genet* 1993, 9(4):138-141.

29. Bishop JM: Cancer: the rise of the genetic paradigm. *Genes Dev* 1995, 9(11):1309-1315.

30. Hahn WC, Counter CM, Lundberg AS, Beijersbergen RL, Brooks MW, Weinberg RA: Creation of human tumour cells with defined genetic elements. *Nature* 1999, 400:464-468.

31. Stratton MR: Exploring the genomes of cancer cells: progress and promise. *Science* 2011, 331(6024):1553-1558.

32. Stratton MR, Campbell PJ, Futreal PA: The cancer genome. *Nature* 2009, 458(7239):719-724.

33. Vogelstein B, Kinzler KW: The Path to Cancer --Three Strikes and You're Out. *N Engl J Med* 2015, 373(20):1895-1898.

## A22 Sphingolipids modulate the fitness of aneuploid cells

### Sunyoung Hwang^1^, Hans Tobias Gustafsson^1^, Ciara O’Sullivan^1^, Aracelli Acevedo-Colina^1^, Xinhe Huang^2^, Christian Klose^3^, Andrej Schevchenko^3^, Robert C. Dickson^2^, Paola Cavaliere^4^, Noah Dephoure^4^, Eduardo M. Torres^1^

#### ^1^Department of Molecular, Cell and Cancer Biology, University of Massachusetts Medical School, Worcester, MA 01605, USA; ^2^Department of Molecular and Cellular Biochemistry and the Lucille Markey Cancer Center, University of Kentucky College of Medicine, Lexington, KY 40506, USA; ^3^Max Planck Institute of Molecular Cell Biology and Genetics, Dresden 01307, Germany; ^4^Department of Biochemistry, Weill Cornell Medical College, New York, NY 10021, USA

##### **Correspondence:** Eduardo M. Torres (eduardo.torres@umassmed.edu)

Aneuploidy is a hallmark of cancer. Importantly, the frequency and degree of aneuploidy correlates with tumor aggressiveness and poor prognosis. Therefore, studying the cellular processes affected by aneuploidy can improve our understanding of its role in tumor biology.

Our previous studies in aneuploid yeast strains revealed that slow proliferation, increased genomic instability and altered metabolism are characteristics shared by aneuploid cells independent of their chromosomal abnormalities. Subsequent studies showed that the aneuploidy-associated phenotypes first discovered in yeast are also present in mouse and human aneuploid cells. This suggests that the cellular responses to aneuploidy are conserved from yeast to humans.

Identifying the cellular processes affected by aneuploidy can reveal how specific genomic alterations help aneuploid cancer cells survive, adapt and thrive despite harboring an abnormal genome.

Due to the negative effects of aneuploidy on cellular physiology and increased genomic instability, selective pressure drives the acquisition of genomic alterations that improve cellular fitness of aneuploid cells. Here, we identified several mutations in genes that regulate sphingolipid synthesis to affect the fitness of aneuploid cells, suggesting that these lipid molecules play an important role in the physiological responses to aneuploidy.

Sphingolipids are synthesized from serine and palmitoyl-CoA. Both long-chain bases (LCBs) and ceramides are sphingolipid intermediates that function as signaling molecules and are rapidly induced upon stress. While ceramides mainly serve to slow cell cycle progression, LCBs activate transcriptional responses and signaling pathways associated with cell wall integrity and survival. Here, we used genetic and biochemical approaches to identify specific sphingolipid molecules that modulate the fitness of aneuploid cells. Transcriptome and proteome analyses of the disomes harboring a mutation that increases LCBs and improves fitness indicate that these molecules regulate membrane protein composition, RNA biosynthesis, and several metabolic pathways that rely on mitochondrial function. Our results provide a better understanding of the physiological role of sphingolipids in controlling the fitness of aneuploid cells. Determining the mechanisms that control the fitness of aneuploid cells can be exploited to target aneuploid cancer cells and to ameliorate the deleterious effects of aneuploidy in Down syndrome or neurodegenerative diseases.

## A23 Efficient transformation of normal human mammary epithelial cells using three pathologically relevant agents does not require gross genomic alterations

### Martha R. Stampfer^1,2^, Lukas Vrba^2^, Mark A. LaBarge^1,3^, Bernard Futscher^2^, James C. Garbe^1^

#### ^1^Biological Sciences and Engineering Division, Lawrence Berkeley National Lab, Berkeley, CA 94720, USA; ^2^The University of Arizona Cancer Center, Tucson, AZ, 85724, USA; ^3^Department of Population Sciences, City of Hope, Duarte, CA, 91010, USA

##### **Correspondence:** Martha R. Stampfer (mrstampfer@lbl.gov)

Most human carcinomas contain genomically unstable cells and express telomerase activity. Widespread instability is first observed *in vivo* at the pre-malignant stage (such as DCIS in breast cancer), and *in vitro* as finite cells with critically shortened telomeres approach replicative senescence. We have proposed that this telomere-dysfunction induced instability is needed to generate the errors required for telomerase reactivation (immortalization), while also generating many additional “passenger” errors that can be carried forward into the resulting carcinomas. Genomic errors that occur prior to immortalization may influence the cancer phenotype and form the basis for ongoing genomic instability in the malignant cells through breakage-fusion-bridge cycles.

We have postulated that immortalization is the rate-limiting step in human carcinogenesis; large organisms such as humans have evolved a very strong barrier to immortalization via stringent repression of telomerase activity in adult cells, presumably to suppress tumorigenesis. Immortalization is crucial for human carcinoma development not only for providing additional proliferative capacity, but also because it makes cells no longer sensitive to oncogene-induced senescence. However, little is known about the cancer-associated immortalization processes. Normal cells from small short-lived mammals like mice do not stringently repress telomerase and lack a significant replicative senescence barrier. Therefore, unlike human cells, murine cells can spontaneously immortalize, and cannot model the immortalization process that occurs during human carcinogenesis. It has been difficult to efficiently immortalize human epithelial cells with pathologically relevant agents in order to examine this process. Immortalization using hTERT precludes examination of the errors responsible for endogenous telomerase reactivation, while viral oncogenes such as SV40T or HPVE6E7 are not etiologic agents for breast cancer and produce many known and unknown effects not seen in breast cancer cells *in vivo*.

We achieved efficient non-clonal immortalization of normal human mammary epithelial cells (HMEC) by directly targeting the two main senescence barriers encountered by cultured HMEC [1,2]. The first barrier - stress-associated stasis - results from stresses maintaining an active Retinoblastoma protein. In human epithelial cells stasis is enforced by elevated p16^INK4A^ levels, and in murine cells, primarily by p19^ARF^ (called p14^ARF^ in human cells). Stasis was efficiently bypassed in HMEC by exposure to shRNA to p16 or overexpressed cyclin D1. Loss of p16 expression and overexpressed cyclin D1 are commonly seen in human breast cancers. The replicative senescence barrier was efficiently bypassed in post-stasis HMEC by MYC transduction; overexpressed or amplified MYC is seen in the majority of human breast cancers. Thus just two pathologically relevant oncogenic agents are sufficient to immortally transform normal finite lifespan HMEC. Further transduction with the mutated ErbB2 oncogene gave anchorage-independent growth. Crucially, these non-clonal immortalized lines, including their anchorage-independent derivatives, exhibited normal karyotypes in early passages. In contrast, all the clonally immortalized lines we have generated (by exposure to chemical carcinogens or other oncogenic agents) display numerous gross genomic abnormalities. These data support our hypothesis that the gross genomic errors and genomic instability present in human carcinomas may not be required *per se* for initiation of transformation, but are needed to generate the errors that overcome tumor suppressive barriers and confer malignancy. Additionally, the genomic instability inherently instigated by telomere dysfunction at replicative senescence may be mainly responsible for initiating the observed cancer-associated genomic instability

This method of efficient step-wise HMEC transformation, in the absence of “passenger” genomic errors, should facilitate examination of telomerase regulation, as well as the role of putative cancer drivers, during malignant progression [3]. We also postulate that the immortalization process –in humans seen *only* in cells that are progressing to cancer - could be a valuable therapeutic target.

**References**

1. Garbe, JC, Vrba, L, Sputova, K, Fuchs, L, Novak, P, Brothman, AR, Jackson, M, Chin, K, LaBarge, MA, Watts, G, Futscher, BW, Stampfer, MR (2014). Immortalization of normal human mammary epithelial cells in two steps by direct targeting of senescence barriers does not require gross genomic alterations. Cell Cycle 13:3423-35. PMID: 25485586

2. Lee JK, Garbe JC, Vrba L, Miyano M, Futscher BW Stampfer, MR, LaBarge, MA (2015). Age and the means of bypassing stasis influence the intrinsic subtype of immortalized human mammary epithelial cells. Frontiers Cell Develop Biol:3:13. PMCID: PMC4356162

3. Vrba, L, Garbe, JC, Stampfer, MR, Futscher, BW (2015). A lincRNA connected to cell mortality and epigenetically-silenced in most common human cancers. Epigenetics 10:1074-83. PMCID: PMC4844203

## A24 Tumor-specific chromosome mis-segregation may be the driving force for tumor recurrence after therapy by restoring tumor heterogeneity

### Yi-Hong Zhou (yihongz@uci.edu)

#### Department of Surgery, University of California, Irvine, CA, USA

**Background**

Tumor heterogeneity with cells having chromosome 7 (Chr7) copy number variation (CNV) or *EGFR* amplification, manifested by double-minute chromosomes (a.k.a. double minutes or DMs), are commonly found in malignant gliomas, especially glioblastoma multiforme. The phenotypic and genetic diversity of tumor subpopulations within a tumor can lead to therapeutic resistance and tumor recurrence. Understanding how tumor heterogeneity is maintained and its equilibrium optimized in re-forming a tumor, after surgery and front-line radiation therapy, should help identify interventions targeted to better combat this deadly disease.

**Materials and Methods**

From established glioma cell lines and fresh GBM specimens, we used two culture conditions (serum adherent and neural sphere cultures) followed by soft-agar colony formation to enrich and clone two key tumor cell subpopulations that may be responsible for the resistance to current therapies [1-3]. We carried out studies on their differential gene/protein expressions, redox and metabolic states, invasion and proliferation phenotypes, with or without acute or partitioned irradiation. Florescence *in situ* hybridization was used to reveal the proportion of cells with Chr7-CNV and EGFR-DM. Tumorigenicities were examined using intracranial xenografts using the subpopulation-enriched cell line or a mixture of the two syngeneic cell lines in various ratios.

**Results**

We demonstrated that aneuploidy of Chr7 (including one copy of Chr7 with 7q deletion), or cells with or without EGFR-DM, characterized the two key tumor cell subpopulations in malignant gliomas, by presenting “go” or “grow” molecular signatures and phenotypes in re-forming orthotropic xenografts [1-3]. The two corresponding tumor cell subpopulations were designated as the stem-like tumor-initiating cells (STIC) and the tumor mass-forming cells (TMC). The lines also differed in redox and metabolic states and in their resistance and response to radiation [2-4]. The heterogeneity of Chr7-aneuploidy was maintained by Chr7 mis-segregation during proliferation of either STIC or TMC to reach an optimal equilibrium that benefited overall tumor growth *in vitro* and *in vivo* [1]. Chr7 mis-segregation can be triggered by therapeutic intervention (e.g. radiotherapy [1]) and alteration in the tumor microenvironment [4]. EGFR-DM heterogeneity, however, was maintained only by cells with DMs, which had the STIC phenotype, and the ability to form colonies comprised of cells without DMs, which then carried the TMC phenotype. Following irradiation, only cells with DMs showed the ability to switch the respiration machinery from glycolic metabolism to oxidative phosphorylation (an anti-Warburg effect), and the molecular-profile from pro-invasive to pro-angiogenic. Furthermore, radiated cells with DMs altered their extracellular microenvironment, not only to promote invasiveness of (unirradiated) surrounding cells with or without DMs, but also to establish an angiogenic micro-environment supporting re-colonization of the (heterogeneous) tumor.

**Conclusion**

Tumor heterogeneity could be maintained by mis-segregation of tumor-specific chromosomes in response to extracellular environmental cues. Radio-resistant glioma cells could form a recurrent tumor with the original tumor heterogeneity by chromosome mis-segregation. New therapeutic intervention by targeting the extracellular compartment to control tumor-specific chromosome mis-segregation may suppress tumor recurrence.

**Acknowledgements**

These studies have received significant scientific contributions from Yuanjie Hu, Chao Ke, Ning Ru, Liping Yu, Yumay Chen, Ping H. Wang, Charles Limoli, and Mark E. Linskey in UC Irvine, Jianhua Xing in the University of Pittsburgh, and Eric R. Siegel in the University of Arkansas for Medical Sciences.

**References**

1. Hu Y, Ru R, Xiao H, Chaturbedi A, Hoa NT, Tian XJ, Zhang H, Chao Ke, Yan F, Nelson J, Li Z, Gramer R, Yu L, Siegel E, Zhang X, Jia Z, Jadus MR, Limoli CL, Linskey ME, Xing J, Zhou YH. Tumor-specific chromosome mis-segregation controls cancer plasticity by maintaining tumor heterogeneity. *PLoS One* 2013;8(11):e80898.

2. Ke C, Tran K, Chen Y, Di Donato AT, Yu L, Hu Y, Linskey ME, Wang PH, Limoli C, Zhou YH. Linking differential radiation responses to glioma heterogeneity. *Oncotarget* 2014;5:1657-1665.

3. Zhou YH, Chen Y, Tran K, Yu L, Linskey ME, Wang P, Limoli P. Symbiosis between GBM cell subpopulations, with or without EGFR amplification, is a new mechanism of tumor resistance to radiation therapy. *Neuro-Oncology* 2015;17:v195-v200.

4. Hu Y, Ke C, Ru N, Chen Y, Yu L, Siegel E, Linskey ME, Wang P, Zhou YH. Cell context-dependent dual effects of EFEMP1 stabilizes subpopulation equilibrium in responding to changes of *in vivo* growth environment. *Oncotarget* 2015;6:30762-72.

## A25 Identification of glioblastoma subpopulations by fluorescence lifetime imaging microscopy (FLIM)

### Andrew L Trinh^1^, Yi-Hong Zhou^2^, Michelle Digman^1^

#### ^1^Department of Biomedical Engineering, University of California, Irvine, CA, USA; ^2^Department of Surgery, University of California, Irvine, CA, USA

##### **Correspondence:** Michelle Digman (mdigman@uci.edu)

Glioblastoma multiforme (GBM) is one of the most aggressive forms of brain cancer. The tumor is known to be composed of heterogeneous subpopulations of cells, potentially playing a role in therapeutic resistance. Therefore, an understanding of the tumor heterogeneity is essential for the development of effective therapies. To further study the tumor heterogeneity, a culture method was developed to isolate distinct subpopulations from an established glioma cell line. These subpopulations include neural stem-like cells and tumor mass cells. Previous studies have demonstrated that these subpopulations have different phenotypes, responses to radiotherapy, and levels of oxidative stress. In this study, fluorescence lifetime imaging microscopy (FLIM) on the metabolic coenzyme, reduced nicotinamide adenine dinucleotide (NADH) was performed. NADH is an essential cofactor for oxidative phosphorylation and glycolysis, therefore the NADH lifetime can be used as an indicator for metabolic states, with longer lifetimes correlating to oxidative phosphorylation and shorter lifetimes correlating to glycolysis. By analyzing the data in the phasor space, the neural stem-like cells and tumor mass cells can be identified using this label-free, live cell, imaging technique. Using this method, the effects of cancer therapies can be evaluated on specific subpopulations of cancer cells to further our understanding of the role of tumor heterogeneity on drug resistance.

